# Inorganic Metal Thiocyanates

**DOI:** 10.1021/acs.inorgchem.4c00920

**Published:** 2024-07-09

**Authors:** Matthew J. Cliffe

**Affiliations:** School of Chemistry, University of Nottingham, University Park, Nottingham NG7 2RD, United Kingdom

## Abstract

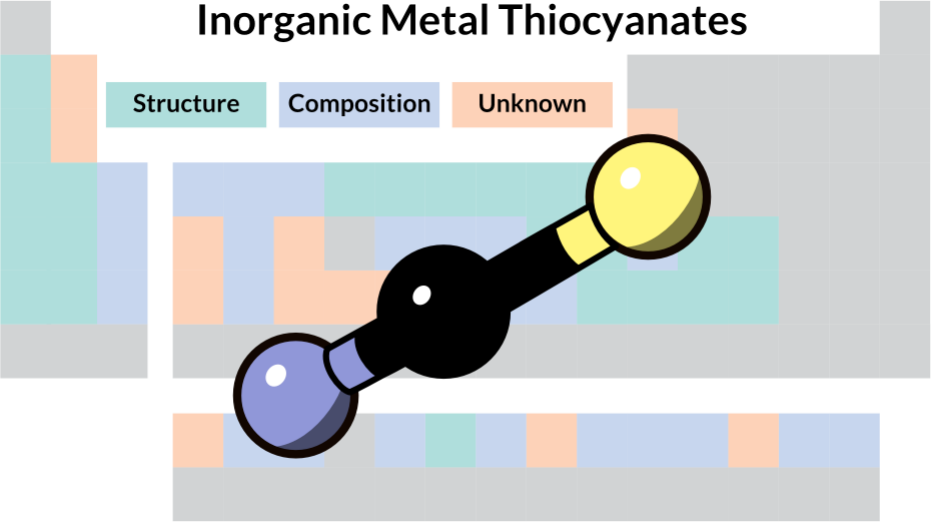

Metal thiocyanates were some of the first pseudohalide compounds to be discovered and adopt a diverse range of structures. This review describes the structures, properties, and syntheses of the known binary and ternary metal thiocyanates. It provides a categorization of their diverse structures and connects them to the structures of atomic inorganic materials. In addition to this description of characterized binary and ternary thiocyanates, this review summarizes the state of knowledge for all other binary metal thiocyanates. It concludes by highlighting opportunities for future materials development.

## Abstract 2

Metal thiocyanates are a diverse family of inorganic materials and some of the simplest coordination frameworks. This review summarizes the structures and chemical and physical properties of all binary and ternary metal thiocyanates, providing a classification for the various structure types and a perspective on the future development of these compounds.

## 1 Introduction

**Figure 1:**
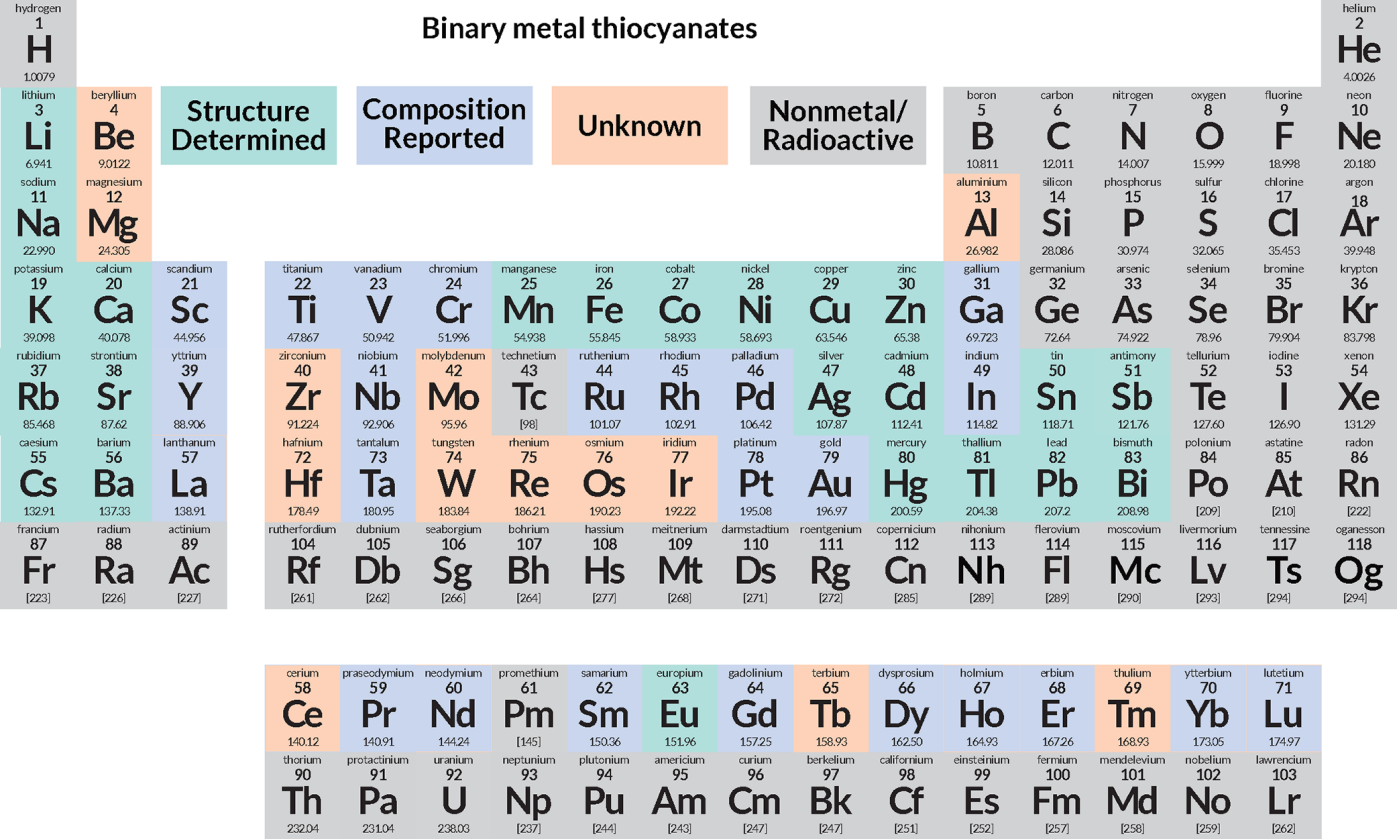
Periodic table of binary metal thiocyanates. The colors highlight whether a metal binary thiocyanate is known with a determined structure (green), the composition has been reported (blue), or there are no reports of a binary structure or where all reports have been disproved (salmon). Elements excluded from this study (nonmetals and radioactive elements) are in gray.

Thiocyanate is a prototypical pseudohalide anion, and it can be found in every area of contemporary chemistry,^1^ most commonly as a ligand in molecular metallocomplexes^2^ but also as an organic functional group,^3^ in biological chemistry as a key metabolite^4^ with potential anticarcinogenic function,^5^ and even in space.^6^ (As is standard, where thiocyanate is bonded purely through N, it has been written as M(NCS)_*x*_ and when through S as M(SCN)_*x*_. In most of the described binary frameworks, thiocyanate is both N and S bound, and for these, M(NCS)_*x*_ is used without implying anything about the local bonding.) The study of thiocyanate has a long history. NCS^–^ was first prepared and isolated by Porrett in 1809, well before the isolation of elemental bromine, fluorine, and iodine.^7,8^ In his pioneering works, Porrett used HNCS to prepare “sulfuretted chyazate” salts (other older trivial names for thiocyanate and its acid include rhodanide, prussous acid, red tinging acid, and sulfocyanide) of a number of elements, including K, Na, Ca, Al, Ba, Sr, Ag, Hg, Cu, Pb, Sn, Bi, Mn, Zn, Co, Ni, Pd, U, Mo, and Cr.^9^ Porrett’s work highlights not only the diversity of thiocyanate chemistry but also how much is yet to be determined about metal thiocyanate chemistry: although these compounds were reported more than 200 years ago, the syntheses and structures of Al, Cr, Mo, Pb, and U thiocyanates have not been confirmed. These open questions in metal thiocyanate chemistry contrast with the “true” metal halides where compounds with nearly every feasible composition have been structurally characterized, from extreme oxidation states (e.g., VCl_5_)^10^ to highly radioactive compounds (e.g., EsCl_3_).^11^ Pseudohalides thus offer synthetic inorganic chemists the rare opportunity to create new (pseudo)binary compounds (Figure 1).

In this review, I describe what is known about the solid-state chemistry of inorganic metal thiocyanates. It presents a systematic overview of their structures and highlights opportunities for developing their chemistry further. The very extensive chemistry of thiocyanate in solution or with coligands has been excluded as it is well-covered elsewhere.^2,12,13^ This review summarizes the chemistry of the binary M(NCS)_*x*_ for every nonradioactive metal and the structure of every ternary metal thiocyanate M_*a*_M_*b*_′(NCS)_*x*_. Special attention has been paid to the structural analogies between metal thiocyanates and other inorganic solid-state compounds. I have relied, in addition to the primary literature, on two book chapters: Golub, Köhler, and Skopenko (1986)^14^ and Williams (1948),^15^ which are the most comprehensive summaries of metal thiocyanate chemistry thus far published. The review begins with a general overview of metal thiocyanates, highlighting characteristic trends. It then moves onto a description of the chemistry of binary metal thiocyanates, describing first the five structural families before summarizing what is known about the binary metal thiocyanates without reported crystal structures. The structures of the ternary metal thiocyanates are then described, being classified first by framework dimensionality and then by characteristic structural features. The review concludes with a brief summary of and prospectus for the chemistry of inorganic metal thiocyanates.^16^

### 1.1 Structural Chemistry

**Figure 2:**
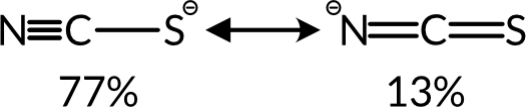
Contributions of the two different resonance forms of NCS^–^ determined using natural resonance theory.^17^

Thiocyanate is a nearly rigid linear anion. The key contrasts in the structural chemistry between thiocyanate and the atomic halides anion arise therefore from the additional degrees of freedom introduced by moving from a sphere with *O*_3_ symmetry to a rod with *C*_*∞v*_ symmetry. The most important new degree of freedom generated is the orientation of the NCS^–^ ligand. This can be parametrized by the M–N–C and M–S–C angles for a bridging thiocyanate ligand. As thiocyanate is stiff, the intramolecular degrees of freedom are less important and deviations from linearity and variations in the bond lengths are typically small, being best described by a resonance structure with a N–C triple bond and C–S single bond (Figure 2).^17^ The orientation of NCS^–^ is so important for the chemistry of thiocyanate because of the different bonding preferences of the N and S termini: the N terminus is harder and typically coordinates well to harder first-row transition metals and s-block metals; the S terminus is softer and most often coordinates to soft metals (late transition metals, 4d and 5d transition metals, and late p-block metals).^18^ This asymmetry means that binary thiocyanates are harder to form, which might otherwise be expected, because often thiocyanate will readily coordinate through one terminus (N or S) to a given metal but only bind weakly through the other end, meaning other ligands (including solvents such as water) can bind more strongly than thiocyanate itself. A common challenge in the synthesis of binary thiocyanates is therefore removing or avoiding competing ligands and coordinating solvents. The large difference in hardness between the N and the S termini offers opportunities for ternary phases, however, and this has been exploited in the synthesis of cation-ordered ternary frameworks (see section 3).^19−22^

**Figure 3:**
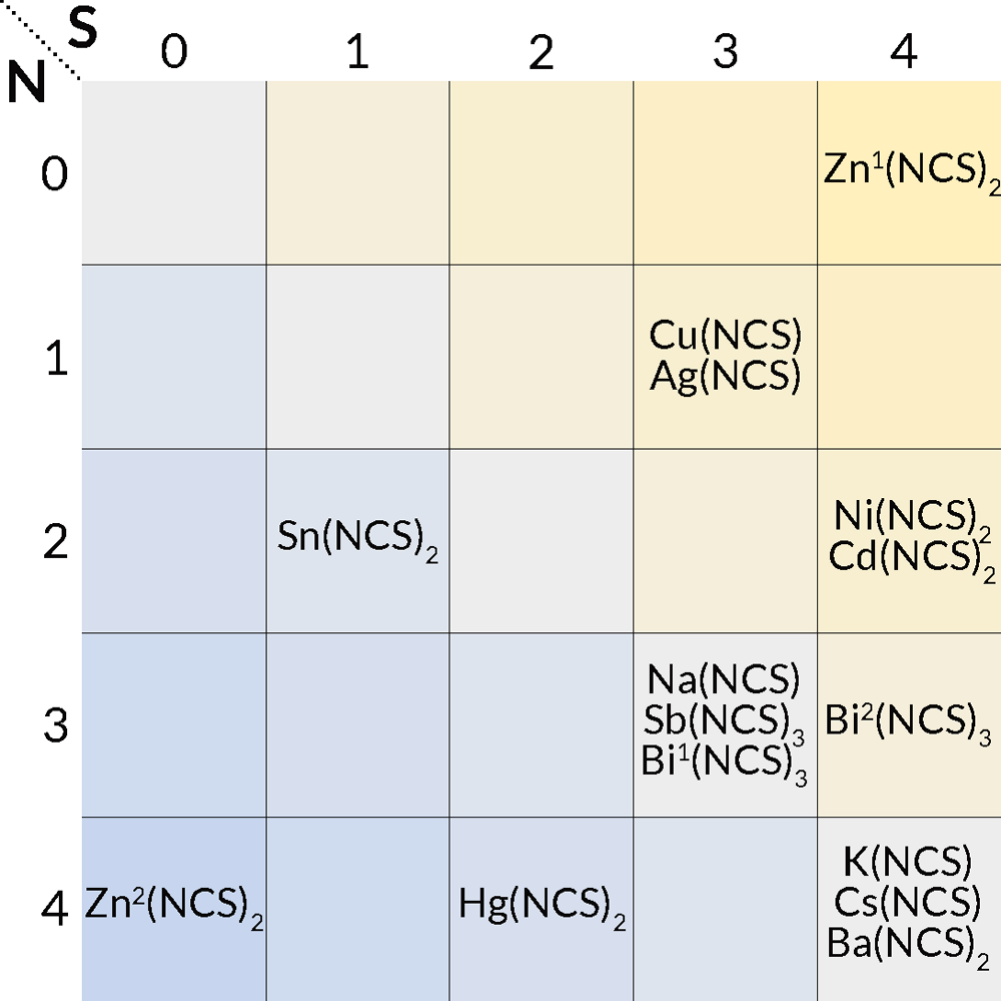
Coordination numbers of the metal in the known binary metal thiocyanates. Where the coordination number could be reasonably larger or smaller, the larger coordination number has been chosen. For structures with sites with different metal coordination numbers, both coordination geometries are listed as M^*n*^(NCS)_*x*_. All reported polymorphic compounds have the same coordination numbers.

The difference in bonding between termini can also be seen in the metal and thiocyanate bond angle: the experimental M–NCS angle distribution peaks at 180°, and the M–SCN angle distribution centers on around 100°,^19^ due to the shapes of the frontier orbitals. This bent M–SCN angle in combination with the lower symmetry of the thiocyanate molecule and the difference in hardness between the N and the S termini show that the symmetries of metal thiocyanates are typically lower those of equivalent halides. The metal azides provide a useful counterpoint, as azide has higher *D*_*∞h*_ symmetry, and indeed KN_3_ is isostructural with the high-temperature phase of KNCS which has head–tail disorder.^23−25^ However, in general, metal azides adopt different structure types as the M–N–M bridging mode is more favorable in azides than thiocyanates.^26,27^ The big difference in bonding preferences between termini in thiocyanate means that the structures are well ordered, aside from highly ionic salts, in contrast to CN^–^ where the pseudosymmetry leads to head–tail disorder.^28−32^ This head–tail order also offers new routes for polymorphism (e.g., section 2.3). A wide variety of coordination modes are adopted by NCS^–^: total coordination numbers vary from two to eight, with S and N coordination numbers going from zero to four (Figure 3). In general, the S terminus tends to adopt higher coordination numbers than the N terminus in the same compound. The differences in local bonding discussed also have consequences for their electronic structures and conductivities.^33^

### 1.2 Function

Metal thiocyanates have diverse functions. The most striking is undoubtedly the strong optical absorption and intense colors which arise largely due to the presence of low-lying metal-to-ligand charge transfer bands, e.g., the well-known blood-red ferric thiocyanate aqueous complex.^34,35^ The optical properties have been most extensively explored in the diamondoid ternaries M[Hg(SCN)_4_] and M[Cd(SCN)_4_] (section 3.2), which have second-harmonic generation (SHG) efficiencies significantly higher than those urea or LiIO_3_, with good UV transparency.^36−40^

Binary thiocyanates containing more reducing metals, such as Cu(I) and Sn(II) (sections 2.3 and 2.5), have appreciable electronic (photo)conductivity.^41−44^ β-CuSCN is a p-type semiconductor with a hole mobility of up to μ = 0.1 cm^2^ V^–1^ s^–1^ ^45^ and an indirect band gap of *E*_g_ > 3.5 eV,^46,47^ leading to good optical transparency in the visible region, though the properties of the two polytypes (2H-β-Cu(NCS) and 3R-β-Cu(NCS)) are not clearly distinguished in many literature reports. These properties together with its synthesis from solution have led to CuSCN being investigated as a transparent hole-transport layer in optoelectronic devices including organic photovoltaics,^48,49^ perovskite solar cells,^50,51^ and light-emitting diodes.^52,53^

The magnetic properties of metal thiocyanates were originally of interest as susceptibility standards due to the relatively temperature-independent magnetic moment of ternary compounds such as CoHg(NCS)_4_. This arises because the large distances between ions produces weak magnetic superexchange and well-isolated ions;^59^ however, long-range magnetic order has been found over comparable distances in Cr[Bi(SCN)_6_],^60^ and even CoHg(NCS)_4_ has evidence of measurable superexchange.^61^ The binary transition metal thiocyanates M(NCS)_2_ have magnetic interactions of similar magnitude^54−58^ to the binary halides (Table 1).^62^ Neutron diffraction studies have determined the magnetic ground states of these binary thiocyanates, showing that Mn(NCS)_2_, Fe(NCS)_2_, and Co(NCS)_2_ share the same stripe-order magnetic ground state,^56^ Ni(NCS)_2_ adopts a layered antiferromagnetic ground state with in-layer ferromagnetic correlations,^56^ and Cu(NCS)_2_ orders with antiferromagnetic correlations along each of the nearest neighbor directions, with the primarily 1D nature confirmed through the significantly reduced ordered moment.^54^ The ternary layered postperovskitoids CsMn(NCS)_3_ and CsNi(NCS)_3_ (section 3.4) both have noncollinear ground states at low temperatures with CsMn(NCS)_3_ adopting a noncoplanar antiferromagnetic structure and CsNi(NCS)_3_ instead showing a noncollinear weak ferromagnetic ground state.^63^ Theoretical investigations suggest that van der Waals metal thiocyanates, Ni(NCS)_2_ in particular, could be of interest as monolayer magnets.^64^

**Table 1:** Summary of the Magnetic Properties of Binary Metal Thiocyanates

	*T*_N_ (K)	*T*_CW_ (K)	References
Cu(NCS)_2_	12(1)	–144.6(5)	Cliffe et al.^54^
Ni(NCS)_2_	54(2)	+29(1)	DeFotis et al.,^55^ Bassey et al.^56^
Co(NCS)_2_	20.0(5)	–44(1)	Shurdha et al.,^57^ Bassey et al.^56^
Fe(NCS)_2_	78.4(3)	–78(3)	Bassey et al.^56^
Mn(NCS)_2_	28.0(3)	–115(3)	Neumann et al.,^58^ Bassey et all.^56^

Inorganic metal thiocyanates are also now being explored as catalysts for a range of reactions, including organophosphate hydrolysis and water photo-oxidation using the perovskitoid M[Pt(SCN)_6_]^65^ and polyurethane foam formation using Sn(NCS)_2_.^66^ The diversity of metal thiocyanate chemistry suggests that other catalytic functions may well be discovered in the future.

## 2 Binary Compounds

There are currently 23 binary metal thiocyanates with reported crystal structures (Figure 1). They can be divided into five categories:

- ionic structures analogous to NaCl and CsCl found for alkali metals and Tl^+^ (section 2.1),
- 3D framework structures with eight-coordinate metals with highly distorted CaF_2_-derived structures (section 2.2),
- van der Waals layered structures with octahedral metals analogous to the NiI_2_ structure type in halide (section 2.3),
- tetrahedral frameworks found for coinage metal monovalent cations related to CuI (section 2.4),
- a variety of complex, 1D, 2D, and 3D frameworks adopted by group 12, 14, and 15 metals (section 2.5).

### 2.1 Alkali Metals

**Figure 4:**
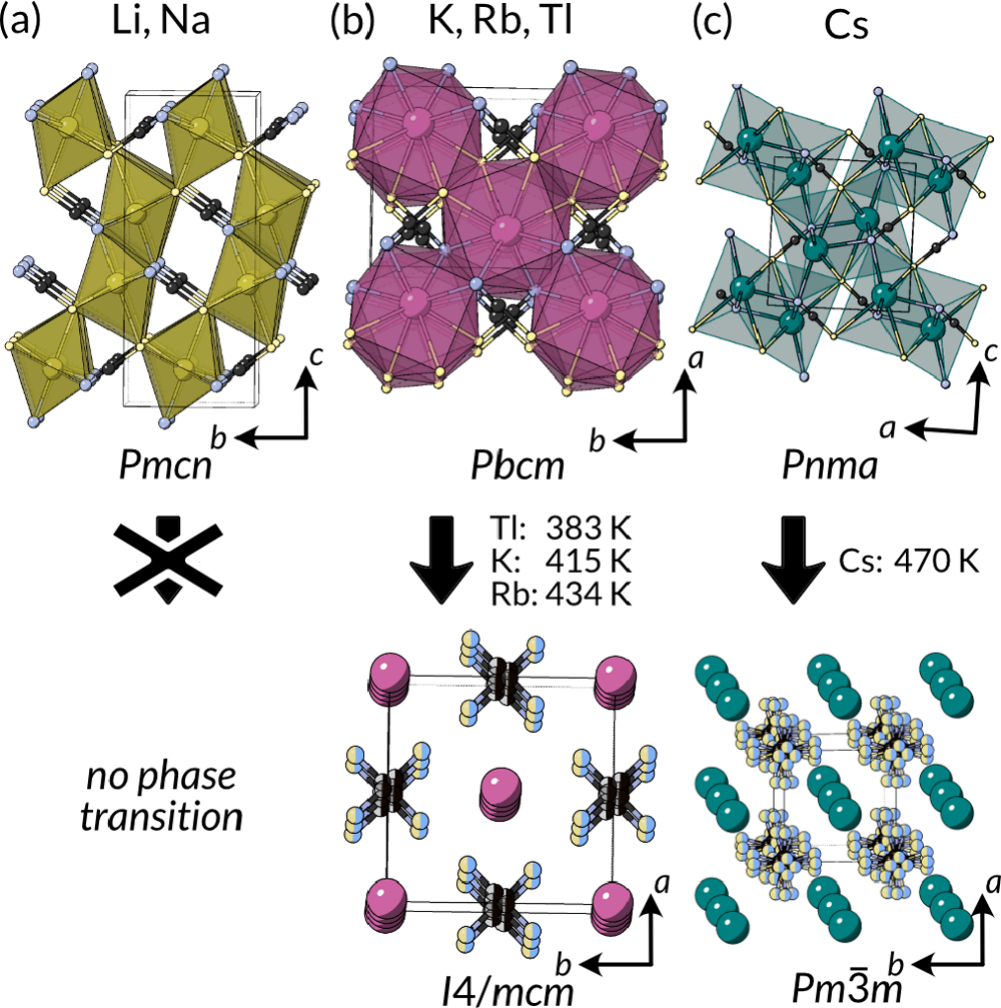
Structures of the alkali metal and Tl(I) thiocyanates, including their phase transitions. (a) Li(NCS) and Na(NCS) adopt rocksalt-derived structures.^67−69^ (b) K(NCS), Rb(NCS), and Tl(NCS) have CsCl-like structures and undergo phase transitions on heating to an anion disordered tetragonal phase.^23,70−72^ (c) Cs(NCS) has a different CsCl-derived structure which transforms into a cubic phase shortly before melting.^73^

The structures and bonding of alkali metal thiocyanates are much more ionic than other metal thiocyanates (thallium has been included in this section due to the similarity of its chemistry) (Figure 4). As metal pseudohalides, they adopt structures similar to the NaCl (Na^+^, Li^+^)^67−69^ and CsCl structures (K^+^, Rb^+^, Tl^+^, Cs^+^).^73−76^ The ionic bonding means that order–disorder transitions are a common feature of these compounds, like other metal pseudohalides, with phase transitions to plastic phases reported for K(NCS), Rb(NCS), Tl(NCS), and Cs(NCS).^23,70−73^

Sodium and lithium thiocyanate both adopt rocksalt-like structures with space group *Pmcn* in which both the N and the S termini are bonded to three metal ions and each metal ion is coordinated by three N and three S atoms.^68,69^ Phase transitions are reported to be absent for Na(NCS) on heating or cooling or on pressure up to 4 GPa.^67^ Lithium thiocyanate is an Li ion conductor due to the presence of Li vacancies in typically synthesized samples.^77,78^

K(NCS), Rb(NCS), and Tl(NCS) all adopt the same ordered orthorhombic *Pbcm* structure at room temperature with eight-coordinate NCS, four through the N terminus and four through the S terminus, and eight-coordinate metal, both in a distorted cubic coordination.^74−76^ On heating, *T*_c_ = 415 K for K(NCS), 434 K for Rb(NCS), and 383 K for Tl(NCS). A high-temperature *I*4/*mcm* tetragonal structure forms, closely related to the low-temperature form but with head–tail NCS disorder.^70,71,75^ The weakly first-order transition has been extensively studied for K(NCS)^23,79^ and is well approximated by a pseudospin model.^80^

NH_4_(NCS) adopts a closely related series of structures to K(NCS).^85,86^ It adopts three phases as a function of temperature. A high-temperature tetragonal phase with dynamic disorder: head–tail thiocyanate disorder accompanied by rotational and positional disorder of the NH_4_^+^ cation above 391 K with an average structure directly analogous to the high-temperature phase of K(NCS).^86,87^ An orthorhombic phase analogous to low-temperature K(NCS) with partial order of both NCS^–^ anions and NH_4_^+^ cations exists between 359 and 391 K.^86,87^ A low-temperature phase with orientally ordered NH_4_^+^ cations, which is also related to the CsCl structure but is structurally quite distinct from the K(NCS) structure^85^

High-pressure thermal analysis and Raman spectroscopy measurements have uncovered phase transitions in K(NCS) at pressure. This suggests that in addition to the room-temperature ambient ordered II phase and high-temperature I phase, there are five additional high-pressure phases up to 10 GPa, though there are as yet no diffraction studies confirming their structures.^88,89^ High-presure studies have also found phase transitions in Rb(NCS) and (NH_4_)(NCS).^89^

Cs(NCS) has, like the other larger metal thiocyanates, a structure related to the CsCl structure with eight-coordinate Cs^+^ and NCS^–^. However, both the low-temperature ordered phase and high-temperature phase are different from the K(NCS)-structured thiocyanates with a more distorted low-temperature *Pnma* phase and a high-temperature *Pm*3̅*m* cubic phase with complete orientational disorder of the NCS^–^ anion.^73^ The order–disorder transition is first order, and the high-temperature cubic phase exists only in a narrow temperature window, between 470 K and its melting point of 479 K. The symmetry relationships between the Cs(NCS) and the K(NCS) structure types, and their phase transitions have been determined by Shlyaykher et al.^75^

**Scheme 1:**
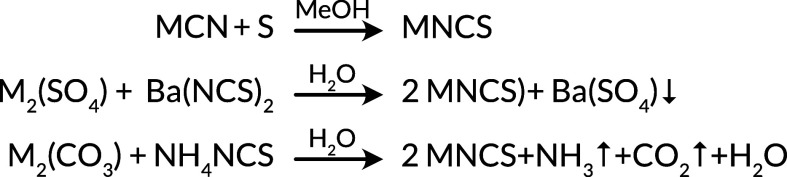
Major Routes for the Synthesis of Alkali Metal Thiocyanates: Li(NCS);^69^ Na(NCS);^81^ K(NCS);^81^ Rb(NCS);^75,82^ Cs(NCS).^83,84^

Na(NCS) and K(NCS) are both readily commercially available but can be made through reaction of the cyanide with sulfur. Rb(NCS) and Cs(NCS) can be prepared by the above route, by reaction of NH_4_(NCS) with M_2_(CO_3_) in water, or by salt metathesis between M_2_(SO_4_) and Ba(NCS)_2_ and filtering off the insoluble BaSO_4_ (Scheme 1).^75,90^ Li(NCS)·H_2_O is also commercially available, and the anhydrate can be prepared through careful dehydration.^69^ Although this review focuses on the solid metal thiocyanates, it is worth noting that the low melting points of ionic metal thiocyanates means that they have significant liquid-state chemistry as molten salts/ionic liquids.^88,91,92^ Recent reports also suggest that solid solutions of Cu(I) and Zn(II) thiocyanates may also melt.^93^

### 2.2 Sr(NCS)_2_

**Figure 5:**
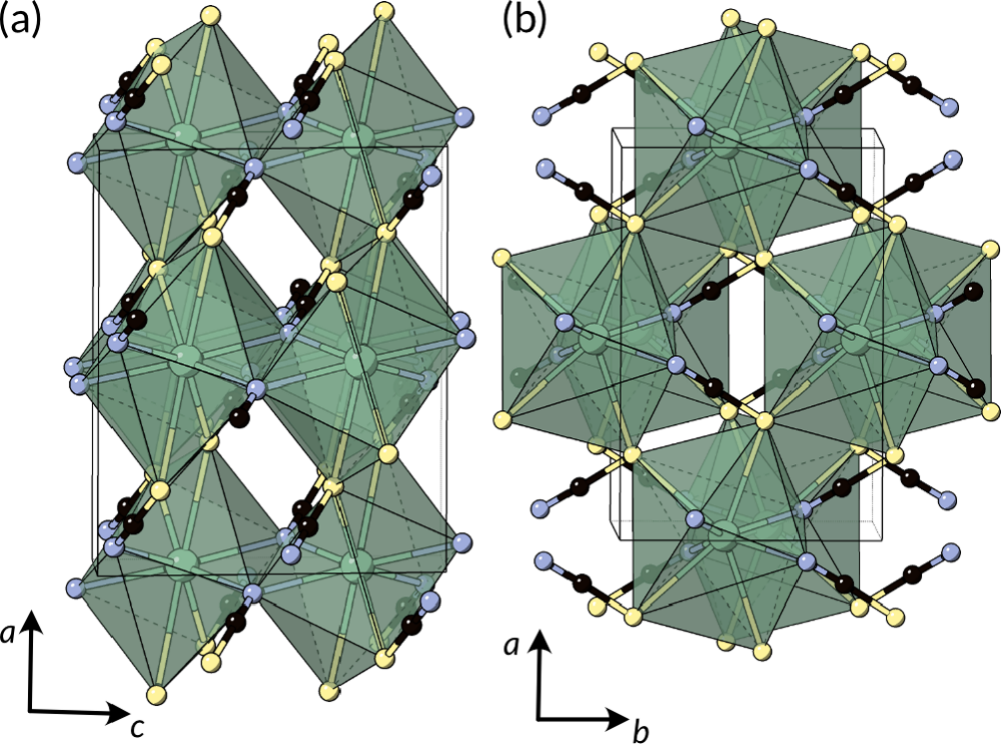
Sr(NCS)_2_ structure type viewed along the (a) *b* and (b) *c* directions, adopted for M(NCS)_2_ where M = Ca, Sr, Ba, Pb, and Eu.^94−96^

**Scheme 2:**
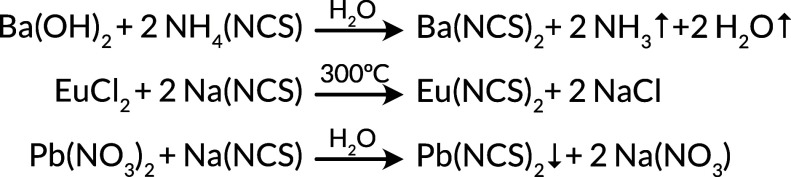
Major Routes for the Synthesis of Sr(NCS)_2_-Type Metal Thiocyanates: (i) Ba(NCS)_2_^82^ and Ca(NCS)_2_;^94^ (ii) Eu(NCS)_2_, Sr(NCS)_2_, and Ba(NCS)_2_;^95^ (iii) Pb(NCS)_2_^98^^,^ The binary compounds can be obtained by dehydration of the hydrates.

The second most common structure type is the 3D Sr(NCS)_2_ structure which forms for the alkaline earths Ca, Sr, and Ba and other large divalent cations such as Pb and Eu (Figure 5).^94−96^ They all crystallize with the *C*2/*c* space group and contain one crystallographically independent M^2+^ cation and NCS^–^ anion. Each M cation is coordinated by four N atoms and four S atoms in a distorted square antiprism. These polyhedra edge share along the *c* axis through the N atoms, and corner share in the *ab* plane. Each NCS^–^ anion is coordinated by four M^2+^ cations in a distorted tetrahedron, and simplification of the structure by considering only the center-of-mass of the NCS^–^ anion reveals a distorted fluorite-type structure. These compounds can be synthesized by reaction between NaNCS and MCl_2_ in sealed glass ampules (Scheme 2). Ba(NCS)_2_, Ca(NCS)_2_, and Pb(NCS)_2_ can be formed through dehydration of the readily synthesisable hydrates.^96,97^ Ba(NCS)_2_·H_2_O is a useful starting material from any thiocyanate that is synthesized in water due to the insolubility of BaSO_4_.

Eu(NCS)_2_ is the only structurally characterized binary thiocyanate containing a paramagnetic ion that does not adopt the Ni(NCS)_2_ structure, though its magnetic properties have not been reported. As an isotypic family, Eu(II) can be doped into the other nonfluorescent member hosts to produce materials with green fluorescence at low temperatures.^99,100^

### 2.3 Ni(NCS)_2_

**Figure 6:**
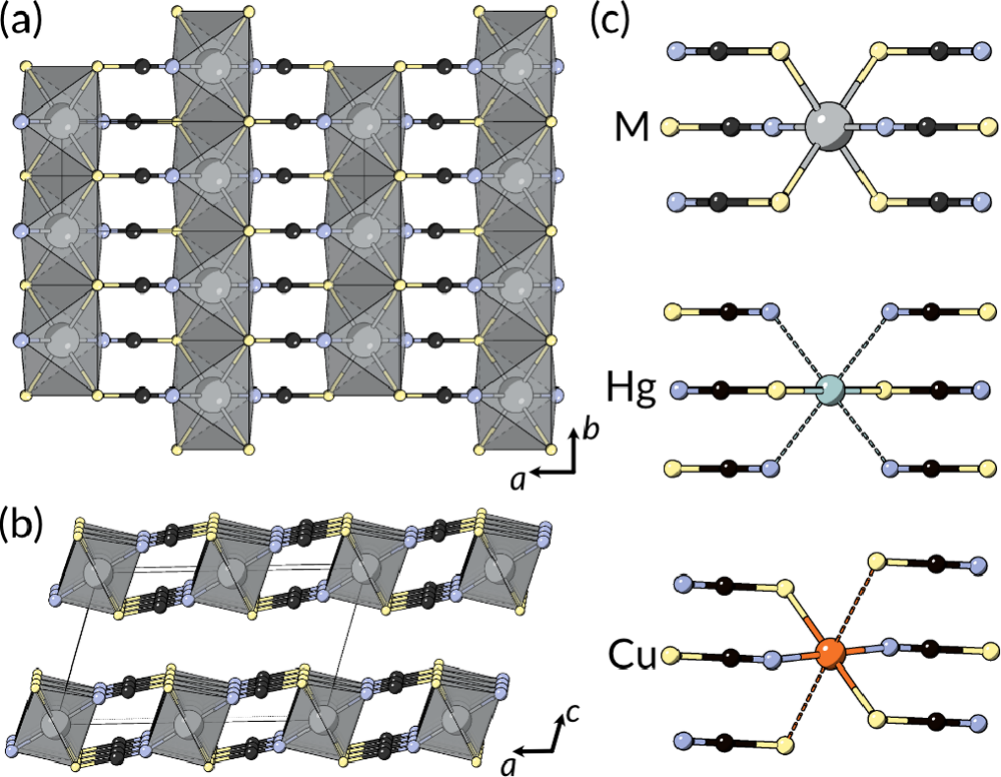
Structures of the transition metal thiocyanates (M = Mn, Fe, Co, Ni, Cu, and Hg): (a) a single *ab* layer viewed along the c* direction and (b) stacking sequence viewed along the [010] direction. (c) Local coordination environment for M = Ni, Co, Mn, Fe; Hg, and Cu. Long bonds are shown by dashed lines.^54,56−58,101−103^

The most common structure type for binary metal thiocyanates is the layered Ni(NCS)_2_ structure, which is found for Ni, Co, Fe, and Mn and with some variation Hg and Cu^2+^ (Figure 6).^54,56−58,101−103^ This structure is analogous to the CdI_2_ structure type common for metal halides,^104^ comprising layers of edge-sharing MN_2_S_4_ octahedra. The neutral layers are held together by van der Waals forces. The aristotype structure crystallizes in space group *C*2/*m*, lowered from the trigonal symmetry of the analogous halides due to the rod-like shape of thiocyanate. The M–SCN angle is around 100°, and the M–NCS angle is roughly 160°. The structures of Hg(II) and Cu(II) deviate from the ideal Ni(NCS)_2_ structure. For Hg(II), the tendency to lower coordination numbers and its soft nature means that there are two shorter Hg–S bonds and four long Hg–N bonds, meaning there is instead HgS_2_N_4_ pseudo-octahedral coordination or nearly linear HgS_2_ coordination if the longer Hg–N bonds are neglected. Cu(II) thiocyanate has a pronounced Jahn–Teller distortion which lengthens one set of Cu–S bonds, lowering the symmetry to *P*1̅ and producing a near one-dimensional structure, and the distortion from the Ni(NCS)_2_ aristotype can be described by the Γ_2_^+^ irreducible representation in Miller and Love’s notation.^105^

**Scheme 3:**
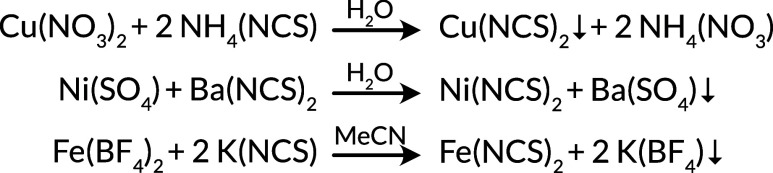
Major Routes for the Synthesis of Transition Metal Thiocyanates: (i) Cu(NCS)_2_^54^ and Hg(NCS)_2_;^106^ (ii) Ni(NCS)_2_,^101^ Co(NCS)_2_,^56^ and Mn(NCS)_2_;^58^ (iii) Fe(NCS)_2_^56^ and Co(NCS)_2_^57^

These compounds can in general be prepared by salt metathesis, either in water from metal sulfate and Ba(NCS)_2_ or in an organic solvent from, e.g., M(BF_4_)_2_ and KNCS, with the insoluble byproduct filtered off and then removal of the solvent from the filtrate containing M(NCS)_2_ (Scheme 3). Typically, this also requires desolvation of a solid solvate with heat or vacuum. Hg(NCS)_2_ and Cu(NCS)_2_ are both insoluble in water and can be prepared by direct reaction of a soluble metal salt and thiocyanate source.^54,106^ The synthesis of Cu(NCS)_2_ can be challenging due to the instability of this compound to Cu(NCS), and it can only be prepared at high concentrations. It will disproportionate on prolonged exposure to water, forming Cu(NCS) and a mixture of byproducts. The difficulty of preparing pure-phase Cu(NCS)_2_ meant its structure was determined 180 years after the first report of its existence.^54,107−109^ Fe(NCS)_2_ is the least chemically stable of the reported Ni(NCS)_2_-structured materials, oxidizing in air, although other compounds are sensitive to moisture: Mn(NCS)_2_ is deliquescent, and Co(NCS)_2_ can be hydrated under humid conditions.^56^ Thermogravimetric analysis suggests that these compounds are thermally stable in inert atmosphere up to approximately 300 °C,^56^ though Hg(NCS)_2_ has long been known for its spectacular thermal decomposition in air to carbonitrides and mercury sulfides and oxides, the so-called “Pharaoh’s Serpent” reaction.^110,111^

These materials are also the only magnetically characterized binary thiocyanates containing paramagnetic ions (there are no magnetic measurements of Eu(NCS)_2_). The first property measurements of Mn(NCS)_2_, Ni(NCS)_2_ and Co(NCS)_2_ included magnetic susceptibility measurements over limited temperature range, which indicated significant antiferromagnetic interactions.^112^ These initial measurements were built upon with further characterization, and long-range antiferromagnetic order has now been found in Ni(NCS)_2_,^55^ Co(NCS)_2_,^57^ Cu(NCS)_2_,^54^ Mn(NCS)_2_,^56,58^ and Fe(NCS)_2_.^56^ Subsequent neutron diffraction revealed unusual layered antiferromagnetism in Ni(NCS)_2_,^56^ and density functional theory calculations suggest that these materials, like the analogous metal halides, may be delaminated to produce 2D magnets.^64^ Despite the propensity for transition metals to adopt a variety of oxidation states, only Cu has fully characterized compounds in an oxidation state other than +2 (section 3.2). Hg(NCS) has been reported to form, and a unit cell has been determined from powder X-ray diffraction.^113,114^ Fe(NCS)_3_ and compounds similar to it have long been investigated due to the utility of the characteristic blood-red color as a chemical test for iron(III),^7^ but isolation of the solid ansolvate has proved challenging.^115^ Slow crystallization of aqueous solution is reported to yield Fe(NCS)_3_, confirmed by iron content analysis,^116,117^ but no structure is known. This family has series of unusual pharmacological functions, acting as both poacher and gamekeeper: Co(NCS)_2_ can be used as a test for the presence of cocaine,^118^ and Cu(NCS)_2_ is a component in salts designed to obscure the presence of the drug.^119,120^

### 2.4 Coinage Metals

**Figure 7:**
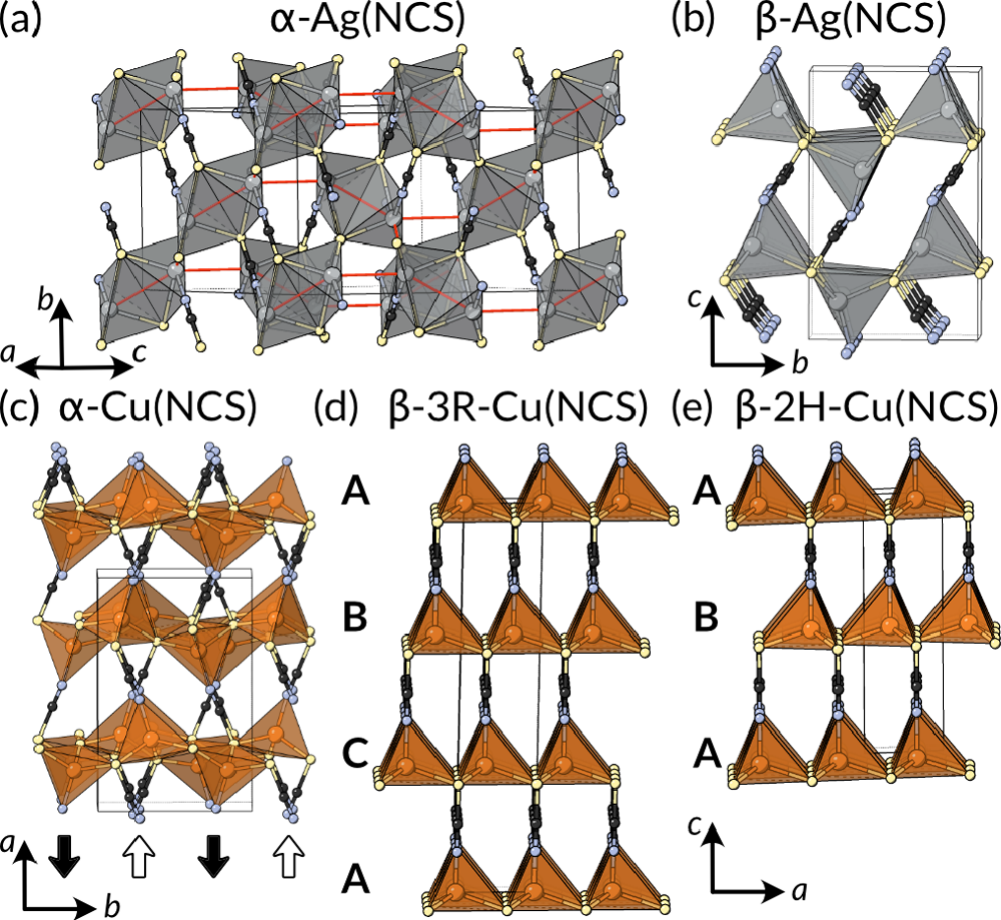
Structures of the polymorphic Ag(I) and Cu(I) thiocyanates. Structures of (a) α-Ag(NCS)-containing Ag_2_ dimers and (b) β-Ag(NCS).^121−123^ Argentophilic interactions highlighted by red lines. Structures of (c) α-Cu(NCS),^124^ (d) β-3R-Cu(NCS), and (e) its polytype β-2H-Cu(NCS).^125,126^

The most polymorphic metal thiocyanates are the monovalent group 10 metals Cu(NCS) and Ag(NCS), which have three and two distinct polymorphs (Figure 7). Cu(NCS) has tetrahedral Cu(I) coordinated by the S and one N atom, and equally, each S atom coordinates to three Cu and each N to one Cu, i.e., NCS^–^ is also tetrahedrally coordinated, like other binary compounds formed from tetrahedrally coordinated cations and anions, which parallels the polymorphism due to different stacking sequences and orientations found in atomic compounds formed from tetrahedrally coordinate metals and anions, such as CuI.^127,128^

There are three reported crystal structures: orthorhombic *Pbca* α-Cu(NCS),^124^*P*6_3_*mc* 2H-β-Cu(NCS), and *R*3*m* 3R-β-Cu(NCS).^125,126^ All of these compounds have in common honeycomb-like CuS layers. In the 2H-β and 3R-β phases, the thiocyanates all point in the same direction, yielding polar structures, and in the α phase, the thiocyanates alternate up and down in a stripe-order pattern, producing a nonpolar structure. The two distinct β polymorphs (which have been confusingly allocated the same Greek letter label) differ only in the stacking of the CuS layers relative to each other, i.e., they are polytypes. 2H-β has AB stacking, analogous to wurzite, and 3R-β has ABC stacking, analogous to sphalerite. The various polymorphs are all slightly soluble in water and can be prepared through various routes, including slow evaporation of solutions of Cu(NCS) in aqueous Na(NCS) solution and the reduction of aqueous Cu(NCS)_2_(NH_3_)_2_ solutions, which requires no additional reducing agent at low concentrations.^124,126^

There are no reports of phase transitions on heating or cooling in Cu(NCS), with powder diffraction measurements from 80 to 400 K on 2H-β showing no phase change and modest thermal expansion (α_a_ = 10.5 M K^–1^, α_c_ = −1.2 M K^–1^).^129^ Indeed, the thermal expansion is considerably smaller than predicted through DFT using the quasiharmonic approximation.^130^ High-pressure Raman spectroscopy and powder X-ray diffraction measurements suggest that the 2H-β phase transforms slowly into α phase above 4 GPa before irreversibly amorphizing through polymerization.^131^ Cu(NCS) is of particular interested as the extended CuS layers allow for significant electronic delocalization and hence p-type transparent conduction.^46,47^ Cu(NCS) is solution processable and has been explored as a hole transport layer in solar cells.^132^

Ag(NCS) has two reported polymorphs.^121−123^ β-Ag(NCS) has a structure related to α-Cu(NCS) and comprises AgS_3_N_1_ tetrahedra connected by thiocyanates into a tetrahedral network. The honeycomb-type AgS layer has alternating up and down thiocyanates.^123^ α-Ag(NCS) contains AgS_3_N_1_tetrahedra but is quite distinct from the other structures. The tetrahedra edge share, and the bond lengths vary significantly. It can alternatively be thought of as consisting of 1D chains running along approximately the [101] direction, analogous to the structure of Ag(CN), connected by longer Ag–S bonds into a 3D network. α-Ag(NCS) has the shortest Ag–Ag distance of 3.28 Å and two further Ag atoms at 3.30 and 3.41 Å, strongly suggestive of significant argentophilic interactions, also common in the silver cyanides.^122,133,134^ The argentophilic interactions form a 3D network with the **srs** or (10,a) topology, and it is likely that these interactions are responsible for the unique structure of α-Ag(NCS). Ag(NCS) is very insoluble and can be prepared by precipitation from aqueous solution from soluble Ag^+^ and NCS^–^ salts.^123^ β-Ag(NCS) is favored by rapid precipitation, acidification, and the presence of gelatin.^123^ A variable-temperature powder neutron diffraction study of the α phase on cooling shows small positive thermal expansion along the *a* direction and larger expansion along the *b* and *c* directions (4.0, 30, and 54 M K^–1^, respectively), but no phase transitions were observed down to 25 K.^135^

### 2.5 Late Transition and p-Block Metals

**Figure 8:**
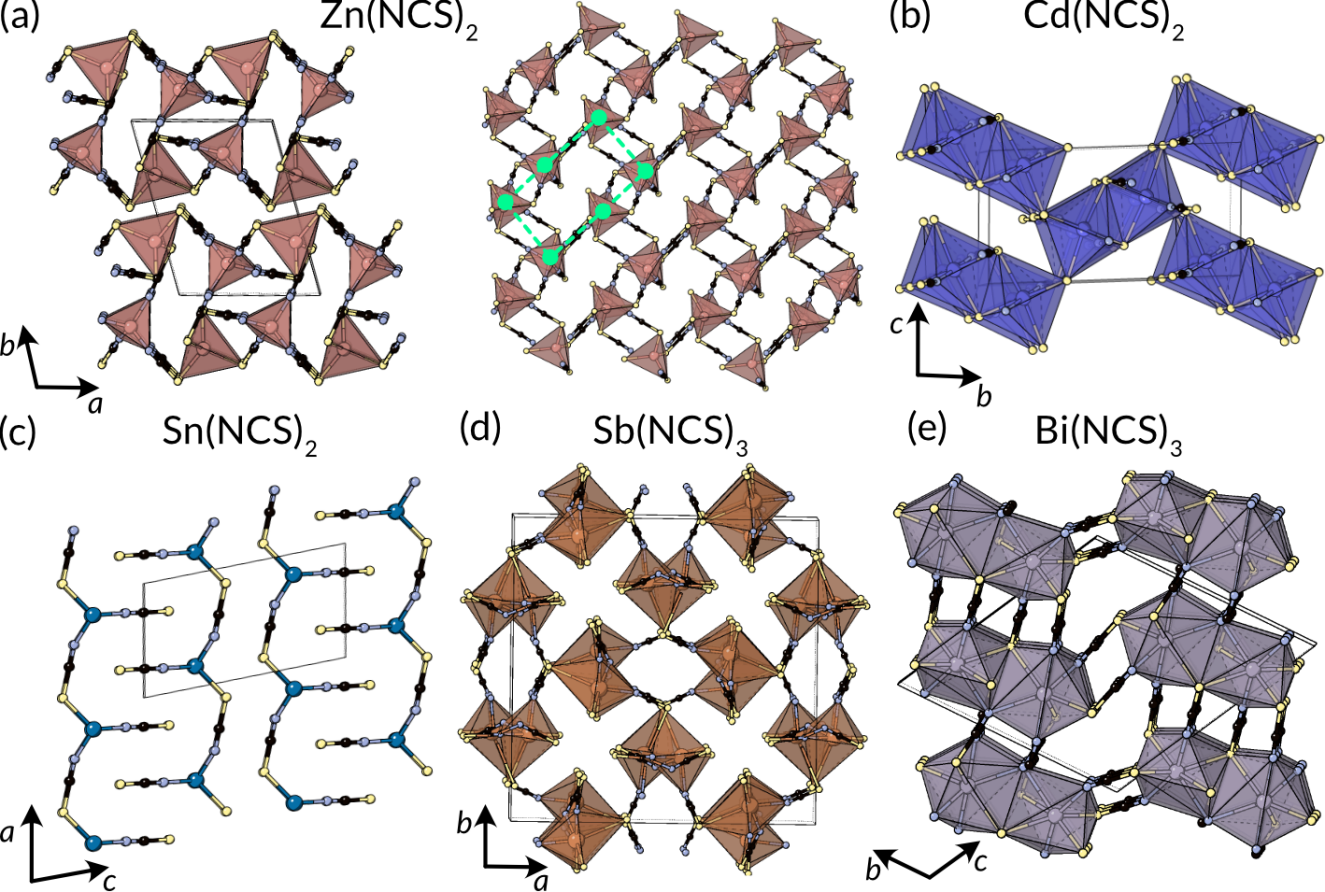
Structures of p-block and late transition metal thiocyanates. (a) Zn(NCS)_2_ viewed along the *c* direction (left) and normal to the *ab* layers.^136^ The 6-rings of the distorted honeycomb are shown in bright green. (b) Ramsdellite-like Cd(NCS)_2_,^137,138^ (c) Sn(NCS)_2_,^66^ (d) Sb(NCS)_3_ viewed along the polar *c* axis,^139^ and (e) Bi(NCS)_3_ with two crystallographically independent Bi atoms.^19^

The remaining binary metal thiocyanates, Sn(NCS)_2_,^66^ Bi(NCS)_3_,^19^ Sb(NCS)_3_,^139^ Cd(NCS)_2_,^137,138^ and Zn(NCS)_2_,^136^ each adopts a unique structure (Figure 8). β-Zn(NCS)_2_ has a tetrahedrally coordinated Zn with two distinct sites, one ZnN_4_ and the other ZnS_4_, and crystallographically independent NCS^–^ anions, each of which bridges two Zn atoms.^136^ This leads to a *P*1̅ van der Waals layered structure, analogous to the SiO_2_ bilayer, where the Zn tetrahedra corner share within a layer to form a honeycomb structure, with the remaining position of the tetrahedra used to join two honeycomb layers into a bilayer.^140^ β-Zn(NCS)_2_ is reported to form by evaporation of aqueous solutions of Zn(NCS)_2_, but on cooling, supersaturated aqueous solutions α-Zn(NCS)_2_ have been reported to form instead.^141^ These two polymorphs have distinct powder diffraction patterns and can also be distinguished by the color of photoluminescence on doping with lead: α-Zn(NCS)_2_ has blue emission and β-Zn(NCS)_2_ has green.^141^ The structure of α-Zn(NCS)_2_ remains unknown, and the potential analogy to SiO_2_ suggests further polymorphism is feasible.

Cd(NCS)_2_ crystallizes in *P*2_1_/*c* and has only a single Cd^2+^ site, octahedrally coordinated by thiocyanate to produce a *trans*-CdS_4_N_2_ coordination polyhedron.^137,138^ Every thiocyanate has the S terminus doubly coordinated and the N terminus singly coordinated. Thus far, the description of Cd(NCS)_2_ is identical with that of Ni(NCS)_2_; however, in Cd(NCS)_2_, there are two distinct NCS^–^ ions and the overall connectivity of the framework is 3D. The structure is thus analogous to the MnO_2_ ramsdellite or Al(OH)O diaspore structure types.^142,143^

Sn(NCS)_2_ crystallizes in *P*1̅ with a prominent stereochemically active lone pair.^66^ There is a single Sn ion, which is three coordinate, SnN_2_S_1_, with one N-bound terminal NCS^–^ and two bridging NCS^–^ anions, producing a 1D coordination polymer. There are three additional longer and weaker Sn···S bonds, which might be considered “tetrel-bonds”, producing a distorted SnN_2_S_4_-coordinated polyhedron and connecting this chain into 2D sheets.^44^ The low symmetry of the structure and the wide variation in bond lengths means alternative descriptions are admitted. Sn(NCS)_2_ appears to be a promising electron-transport layer for photovoltaic devices^144,145^ Tin(II) thiocyanate crystallizes from an aqueous solution of Sn(SO_4_) and Na(NCS), whereas Zn(NCS)_2_ and Cd(NCS)_2_ can be prepared by salt metathesis between the metal sulfate and Ba(NCS)_2_ in water.

Bi(NCS)_3_ can be synthesized as orange-yellow crystals through the slow evaporation of aqueous H_3_[Bi(SCN)_6_] solutions obtained by the acid–base reaction between ethereal HSCN and Bi_2_O_2_(CO_3_).^19^ Like Sn(NCS)_2_, it also has stereochemically active metal ions, crystallizing in *P*1̅; however, it is the most structurally complex of all of the reported metal thiocyanate binaries, with two distinct metal ions and six distinct NCS^–^ ions.^19,146^ Both bismuth sites have a distorted coordination geometry; Bi1 has four short bonds, BiN_2_S_2_, but including longer bonds can be considered to be seven coordinate, BiN_4_S_3_; Bi2 considering only short bonds is a very distorted octahedron, BiN_2_S_4_, but has an additional short contact to a third N atom to make a seven-coordinate BiN_3_S_4_ coordination geometry. These larger (seven vertex) polyhedra directly edge share through one nitrogen and one sulfur and are connected into a 3D framework through the length of the thiocyanates.

Sb(NCS)_3_ can be obtained as colorless crystals by removing THF from the relevant solvate, itself synthesized from the reaction of SbF_3_ and trimethylsilyl isothiocyanate.^139^ It crystallizes in the polar *I*4_1_*cd* space group, again with a very significant stereochemically active lone pair. Considering only the shortest bonds, it can be thought of as comprising solely of molecular Sb(NCS)_3_ units. If slightly longer (3.1 Å) Sb–S bonds are considered, the Sb(III) cation has a distorted octahedral coordination geometry, SbN_3_S_3_. These octahedra edge share (through the length of the thiocyanate) to form infinite chains. These chains corner share (though only on alternate layers) to form a 3D network. For these main group and d^10^ transition metal elements, the lack of crystal-field stabilization energy appears to permit a much wider variety of structure types, and indeed, the presence of significant stereochemically active lone pairs in Sn(II), Sb(III), and Bi(III) produces the most complex reported binary metal thiocyanate structures. Notably, Pb(II) crystallizes in the Sr(NCS)_2_ structure type without a stereochemically active lone pair.

### 2.6 Missing Binary Thiocyanates

The above compounds are the 23 binary metal thiocyanates with reported crystal structures. Nearly all stable (and some radioactive) metals have been reported to form complexes with thiocyanate,^2^ and it is likely stable binary metal thiocyanates can also be formed for nearly all metals too. Indeed, 24 further metallic elements have been reported to form binary thiocyanates, leaving only 13 nonradioactive metallic elements with no reports at all (Figure 1). The 37 binary metal thiocyanates without reported structures are likely challenging to synthesize in part due to the need to simultaneously bond both ends of the thiocyanate with their different hardnesses and the propensity to form stable solvates rather than extended frameworks. Below, I briefly outline the missing 37 binary metal thiocyanates and where their synthesis and composition has been reported, the current state of knowledge. The lack of full characterization for many of these compounds means that these reports should be treated with caution. Even taking into account compounds without complete characterization it is notable that there are currently very few reports of variable oxidation states in metal thiocyanates, despite the range of oxidation states found in the metal halides.

The first class of missing binaries is those formed from hard, oxophilic metals, where although M–NCS bonds are facile to form, M–SCN is not competitive with most solvents. There are two missing group 2 thiocyanates, Be(NCS)_2_ and Mg(NCS)_2_. Beryllium thiocyanate complexes are known,^147^ but preparation of the anhydrous solid Be(NCS)_2_ has rarely been attempted and appears to be challenging.^148^ Mg(NCS)_2_·*x*H_2_O has been extensively studied, and investigation of its dehydration suggests that preparation of the anhydrate from the hydrate is not feasible and previous reports of Mg(NCS)_2_ were erroneous.^149^ Likewise, the oxophilicity of group 3 and lanthanide elements means hydrates are readily formed. Attempts to synthesize Sc(NCS)_3_ from solution produce solvates of stoichiometry Sc(NCS)_3_L_3_, though a poorly characterized “yellow scandium trithiocyanate” has been hinted at through desolvation of the ethereal solvate.^150,151^ Other group 3 and lanthanide thiocyanates M(NCS)_3_, M = Y, La, Pr, Nd, Sm, Gd, Dy, Ho, Er, Yb, and Lu, have been synthesized by slowly dehydrating the hydrates using reduced pressure (100 Torr) at 50 °C with the presence of P_2_O_5_ over 1 week, with compositions determined via elemental analysis.^152^ Other lanthanide thiocyanates might be prepared by dehydrating the metal thiocyanate solvates in the same way.^153^

Moving right along the periodic table, Ti(NCS)_4_ has been reported to be synthesized by the desolvation of THF solvates at 150 °C under vacuum, producing a red-black crystalline powder. Analysis of the IR spectrum of Ti(NCS)_4_ suggests octahedrally coordinated Ti cations with at least some bridging NCS^–^ anions.^154^ These data thus suggest that the structure might be more similar to TiF_4_, a 1D structure comprising corner-sharing TiF_6_ octahedra,^155^ than the later halides TiX_4_, X = Cl, Br, and I, which are all tetrahedral molecular materials with no Ti–X–Ti bridges.^156−158^ There are no reports of Zr(NCS)_4_ or Hf(NCS)_4_, though the DMF solvates of both are reported.^159^ It is worth noting that one of the few ways to readily separate Zr and Hf is by preparing the thiocyanato oxohydroxy metal complexes, and so it is possible that Zr(NCS)_4_ and Hf(NCS)_4_ will have different structures and chemistries.^160^

Binary thiocyanates are attested for all group 5 metals, with the most thoroughly investigated being V(NCS)_3_, which can be made through salt metathesis in diethyl ether between NaNCS and VCl_3_, producing a red-brown powder.^161^ V(II) and V(IV) thiocyanate complexes are also stable, though only solvates have been reported.^162,163^ Nb(NCS)_5_ and Ta(NCS)_5_ are reported to be dimers on the basis of IR spectroscopy and were prepared by desolvation of the acetonitrile solvates.^164^

Group 6 binary thiocyanates are less well understood. Cr(NCS)_2_L_2_ is well known for a range of solvents, but the ansolvate has not proved possible to prepare, likely in part due to the high sensitivity of Cr(II) to oxidation.^57,165^ Cr(NCS)_3_ is reported to be a deliquescent dark green powder, though characterization is scant.^15^ A variety of Mo(V) and W(VI) thiocyanate solvates can be made through the reaction of K(NCS) or Na(NCS) with MoCl_5_ and WCl_6_ in organic solvents, forming dark red (Mo) or dark brown (W) crystals, but desolvation was not possible by applying a vacuum.^166^ Oxothiocyanato metal complexes are common in these high oxidation states,^167−169^ but lower oxidation state molecular molybdenum and tungsten thiocyanate complexes with coligands and/or additional countercations are common and can include metal–metal bonding.^170−172^ DFT investigations of the binary Mo(NCS)_2_ and W(NCS)_2_ suggest they are stable and adopt layered structures, analogous to the Hg(NCS)_2_ structure, though with trigonal prismatic metal coordination and metal–metal bonding.^173^ The computational stability of these hypothetical layered structures relative to cluster structures, e.g. a M_6_(NCS)_12_ hexametallic cluster, is not reported, despite these being the stable structures of the analogous halides.^174^

The 3d late transition metals all form divalent binary thiocyanates with the Ni(NCS)_2_ structure, as described above, though other oxidation states are unknown except for hints of Fe(NCS)_3_.^116,117^ The late 4d- and 5d-block binary metal thiocyanates are, in contrast, much less well understood. Technetium and rhenium thiocyanate complexes are known in a variety of oxidation states,^175,176^ but there are no reports of the binary compounds aside brief discussion of an orange-red solid complex formed on reaction of ReO_4_ with KNCS and SnCl_2_, of probably composition Re(NCS)_4_ or ReO(NCS)_4_.^177,178^

Rh(NCS)_2_ is reported to be a brown powder formed by the reaction of Rh_2_(CCl_3_COO_2_)_4_ dimers with potassium thiocyanate, which is insoluble in water, though the presence of an infrared absorption band at 1614 cm^–1^ suggests perhaps more extensive reaction than simple salt metathesis has occurred.^179^ The synthesis of Rh(NCS)_3_ and Ru(NCS)_3_ as orange-red (Rh) and dark brown (Ru) powders from the salt metathesis in acetonitrile between K(NCS) and the metal trihalide has been reported.^180,181^ There are no reports of the synthesis of binary Os(NCS)_*x*_ or Ir(NCS)_*x*_. As both [Os(SCN)_6_]^3–^ and [Ir(SCN)_6_]^3–^ anions exist, if the ansolvates exist, *x* is most likely to be three.^182,183^ I note that Os(NCS)_3_ is likely to be the best candidate for a magnetic ReO_3_-structured metal thiocyanate as it has the most extensive linkage isomerism with thiocyanate of any paramagnetic metal.^184^

Pd(NCS)_2_, Pt(NCS)_2_, and Au(NCS) have all been reported, and chemical elemental analysis suggests they are pure-phase compounds. Pd(NCS)_2_ and Pt(NCS)_2_ are reported to be formed by the thermal decomposition of M(NCS)_2_(NH_3_)_2_.^185,186^ Aurous thiocyanate has also been reported to form as a white powder by thermal decomposition of the organometallic complex [Et_2_Au(NCS)]_2_ through addition to hot xylene (125 °C). ***Warning:** decomposition of the solid [Et_2_Au(NCS)]_2_ at temperatures above 80 °C is reported to be explosive*.^187^ Au(NCS)_3_ has been reported to form from the reaction of excess AuCl_3_ with soluble alkali metal thiocyanate salts, though further details of the synthesis and characterization are lacking.^15^

The only crystal structure of a group 13 binary thiocyanate is Tl(NCS); however, many more should be thermodynamically stable. A number of routes for the synthesis of In(NCS)_3_ have been reported: salt metathesis in ethanol between Na(NCS) and InCl_3_; metathesis in water between Ba(NCS)_2_ and In_2_(SO_4_)_3_; reaction between In metal and Hg(NCS)_2_ and sulfuration of In(CN)_3_.^188,189^ Analysis of X-ray powder diffraction measurements suggests a cubic cell of 12 Å, which implies a ReO_3_-type model consisting of alternating InN_6_ and InS_6_ octahedra, though there is no atomistic structural model or quantitative refinement of the diffraction data.^188^ This structure would be related to the observed CsCd(NCS)_3_ structure (section 3.1).^188^ An analogous route to create Ga(NCS)_3_, that is the aqueous metathesis reaction between Ba(NCS)_2_ and Ga_2_(SO_4_)_3_, produced a hydrate with N-bound thiocyanate which could not be further dehydrated.^190^ The formation of a stable N-bound thiocyanate hydrate is also reported for aluminum, though again desolvation was not possible.^191^ The potential for Tl(III) or In(I) thiocyanates has not been extensively explored, though the aqueous reaction of TlCl_3_ with KNCS has been reported to oxidize NCS^–^ to HCN and H_2_SO_4_.^192^

In group 14, Pb(NCS)_2_ and Sn(NCS)_2_ are well known, but group 14 metal thiocyanates with +4 oxidation state should also be feasible. Sn(NCS)_4_ is reported to form from the reaction of thiocyanogen (NCS)_2_ and Sn(OH)_4_ in ethereal solution^193^ though without any further characterization.

## 3 Ternary Metal Thiocyanates

In addition to the binary metal thiocyanates outlined above, there are many more solid-state inorganic metal thiocyanates with more complex compositions. In this review I have restricted discussion to the ternary homoleptic thiocyanates, A_*x*_M_*y*_(NCS)_*z*_, as they illustrate the distinctive features of metal thiocyanate chemistry. Ternary thiocyanates can be broadly classified by the dimensionality of the coordination framework: 3D-, 2D-, and 1D-connected frameworks and 0D salts containing only discrete metal thiocyanate complexes.

### 3.1 3D: Perovskitoid

There are two main families of 3D frameworks which can be realized for a wide range of compositions: perovskitoid, based on octahedrally coordinated metals, and diamondoid, based on tetrahedral metals. In common with other metal thiocyanates, the difference in chemistry between the N and the S termini is key and means that in both cases “double” structures with alternating cation order predominate. Indeed, all reported metal thiocyanate perovskitoid and diamondoid structures contain alternating MN_*n*_ and MS_*n*_ polyhedra. This means most of these materials use two different framework-forming metal ions: a harder metal for the MN_*n*_ polyhedron and a softer metal for the MS_*n*_ polyhedron.

**Figure 9:**
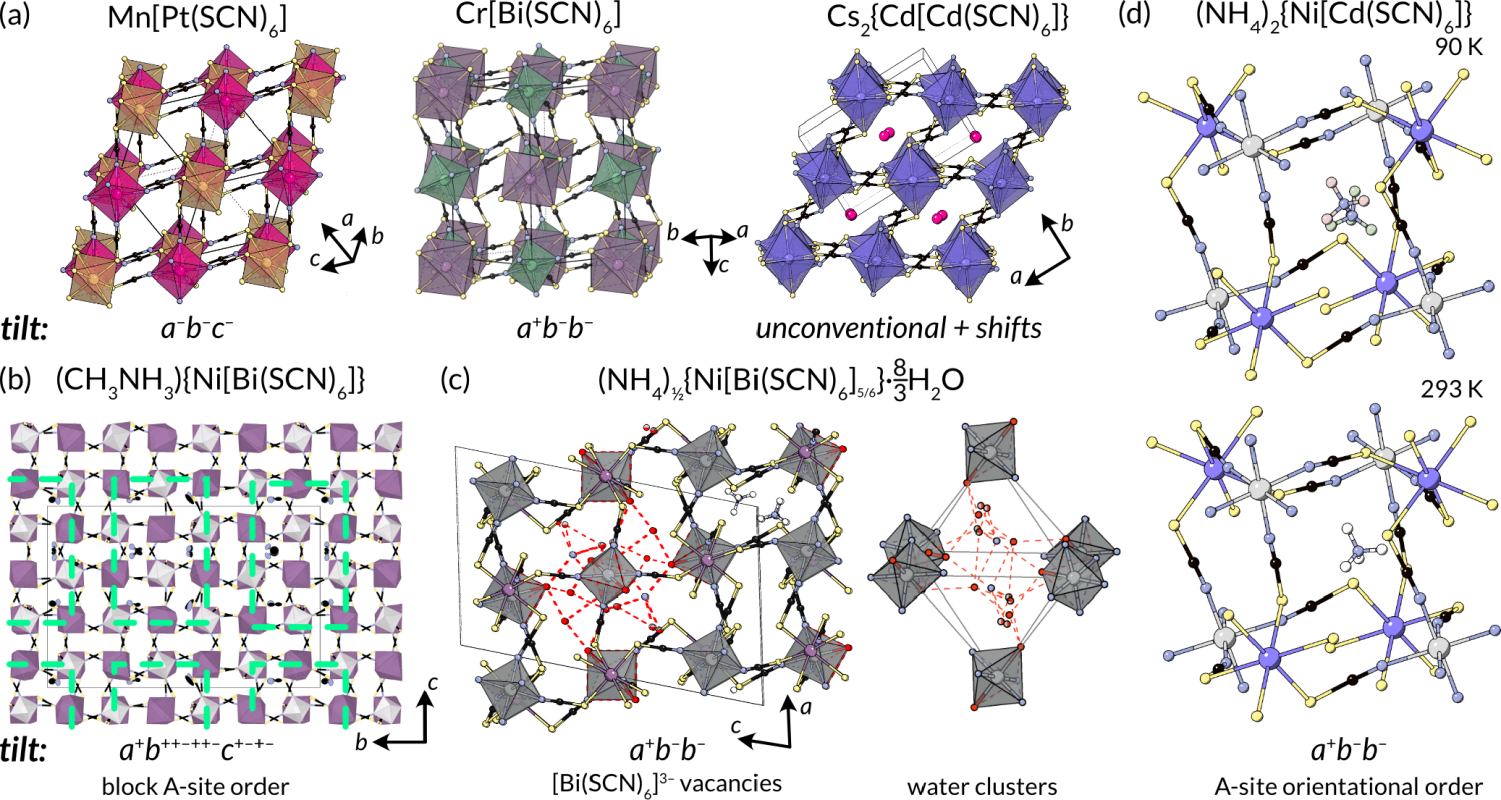
Structures of perovskitoid thiocyanates A_*x*_{M[M′(SCN)_6_]}, all with rock-salt M site order. (a) The three classes of perovskite, with M′ = Pt^4+^, Bi^3+^, and Cd^2+^.^19,65,83^ (b) Complex A-site cation order in (CH_3_NH_3_){Ni[Bi(SCN)_6_]}.^194^ (c) Ordered [Bi(SCN)_6_]^3–^ in (NH_4_)_1/2_{Ni[Bi(SCN)_6_]_5/6_·(8/3)H_2_O.^195^ (d) A-site cation orientational order transition in (NH_4_)_2_{Ni[Cd(SCN)_6_]}.^196^

The perovskitoid structures comprise corner-sharing octahedra linked by a single μ_1,1_-coordinated thiocyanate, potentially with cations contained with the pseudocubic cavity (Figure 9). Like the nonmolecular perovskitoid analogues, these metal thiocyanates are an extremely diverse family. I have also included here some perovskitoids of more complex stoichiometry to situate the chemistry of these materials within the broader family and highlight some of the unique structural features. The limited range of softer metals means currently known NCS-perovskitoids can be classified into three families by the identity of the MS_6_ cation: A_*x*_M[Bi(SCN_6_)_*y*_],^19,194,195^ A_*x*_M[Pt(SCN)_6_],^22,65^ and A_*x*_M[Cd(SCN)_6_].^83.196,197^ The occupancy of the A-site cation varies from *x* = 0 (i.e., an ReO_3_ or Prussian Blue type structure^198^) to *x* = 2, the standard perovskite stoichiometry.

The very first known perovskitoids were M[Pt(SCN)_6_], M = Fe, Cu, and Co, reported by Buckton in 1856,^22^ though the stated stoichiometry was wrong due to the incorrectly determined masses of N and S at the time, and the lack of structural characterization techniques available at the time precluded an understanding of its perovskitoid structure. Recent work has determined the structures of not only these compounds but also M = Mn and Ni.^65^ Although Buckton’s initial report was understandably lacking in diffraction-based characterization, it did include the, unlikely to be replicated, thorough gustatory characterization, highlighting the “exceedingly nauseous taste” of soluble [Pt(SCN)_6_]^4–^ compounds. In addition to the perovskitoid-structured compounds, Buckton also reported K_2_[Pt(SCN)_6_] (Buckton’s salt), now partially structurally determined (section 3.6),^199^ and otherwise unknown compounds of [Pt(SCN)_6_]^4–^ with Hg, Ag, Pb, and Au. Other platinum-based perovskitoids are very likely feasible.^200^

The first metal thiocyanate perovskitoid to have its structure determined was CsCd(NCS)_3_. Rather than having distinct hard and soft cations on the MN_6_ and MS_6_ sites, it has Cd ions on both. The Cs^+^ cation resides in the pores of the anion framework.^83^ Larger cations can also be incorporated within this framework, as shown by [(CH_3_)_3_S]_2_{Cd[Cd(SCN)_6_]}.^197^ Cadmium thiocyanate perovskitoids can also occur with mixed metals, where the MN_6_ site is occupied by a harder first-row transition metal, including A_2_{Ni[Cd(SCN)_6_]} A = K, NH_4_, (CH_3_)_2_NH_2_, and [(CH_3_)_2_NH_2_]_2_{Mn[Cd(SCN)_6_]}.^196,197^

The use of Cr[Bi(SCN)_6_] to quantitatively analyze the presence of bismuth in solution dates from the 1930s,^201^ but its structure was determined only recently together with the isostructural Sc[Bi(SCN)_6_] and Fe[Bi(SCN)_6_].^19^ Other bismuth-based perovskitoids containing divalent transition metals and A-site cations are now known, including both A{Ni[Bi(SCN)_6_]}, A = K, NH_4_, and CH_3_NH_3_, and more complex stoichiometries: C(NH_2_)_3_{Ni[Bi(SCN)_6_]_5/6_}, Mn_2_Bi(NCS)_7_·7H_2_O, Co_9_Bi_6_(NCS)_36_·38H_2_O, and M_6_Bi_5_(NCS)_30_·16H_2_O·3NH_4_ M = Ni, Co.^194,195^

As perovskitoids, multiple orderings lowering the symmetry from *Pm*3̅*m* naturally arise. Rocksalt “M”-site order is strongly enforced in all known NCS perovskitoids due to the preference for MS_6_ and MN_6_ octahedra. The fact that the S terminus binds in a bent fashion imposes large cooperative tilts, meaning that NCS perovskitoids have tilts along every axis, which combined with the presence of the rocksalt order means only six simple tilt sequences are possible in the Glazer notation *a*^+^*a*^+^*a*^+^, *a*^+^*b*^+^*c*^+^, *a*^–^*a*^–^*a*^–^, *a*^–^*b*^–^*c*^–^, *a*^+^*b*^–^*b*^–^, and *a*^+^*a*^+^*c*^–^.^202,203^ Of these, the M[Pt(SCN)_6_] perovskitoids adopt the *a*^–^*b*^–^*c*^–^ sequence, and most M[Bi(SCN)_6_] and M[Cd(SCN)_6_] compounds adopt the *a*^+^*b*^–^*b*^–^ sequence.^19^ As the sulfur bond angle guarantees a tilted structure, larger cations (e.g., molecular cations) induce complex tilts, e.g., CH_3_NH_3_{Ni[Bi(SCN)_6_]} adopts the *a*^+^*b*^++–++–^*c*^+–+–^ tilt or cation vacancies, e.g., C(NH_2_)_3_{Ni[Bi(SCN)_6_]_5/6_}, to accommodate them.^194^ The greater coordinative flexibility of perovskites with Cd(NCS)_6_ or Mn(NCS)_6_ octahedra allows for unconventionial tilts and columnar shifts (a degree of freedom impossible in nonmolecular perovskites),^204−206^ particularly in the presence of larger cations, whether molecular or Cs^+^.^83,197^ (CH_3_)_3_S)^+^ is the largest “A”-site cation reported in a thiocyanate perovskitoid. When even larger cations are used they cannot be included within the cages and nonperovskite phases start to form, most typically phases containing 1D face-sharing chains, analogous to the CsNiCl_3_ structure type.^207−209^

There are also potential orderings associated with the A site. In general, molecular A-site cations are orientationally ordered due to the low symmetry of the frameworks,^194^ but an order–disorder phase transition has been found in (NH_4_)_2_{Ni[Cd(SCN)_6_]},^196^ and A sites are often disordered in frameworks containing B-site vacancies.^194,195^ In perovskites with partial occupancy of the A site, the tilts strongly couple to the A-site order, producing complex orders not found in nonmolecular perovskites, which have the potential to produce electrical polarity.^194^

### 3.2 3D: Diamondoid

**Figure 10:**
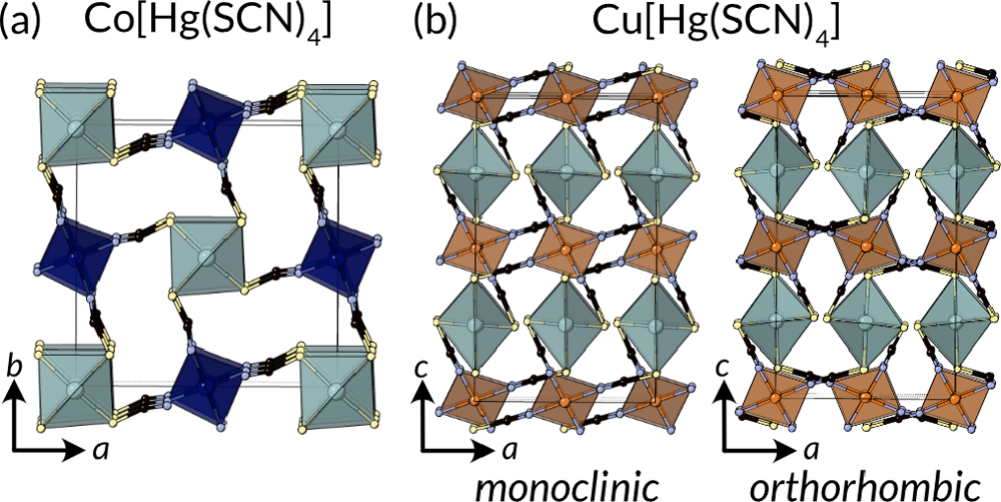
Structures of (a) diamondoid Co[Hg(SCN)_4_]^20^ and (b) polymorphs of Cu[Hg(SCN)_4_]: monoclinic phase with **pts** topology; orthorhombic phase with 4,4T3 topology.^210,211^

For tetrahedral metals bound by thiocyanate, the 1:1 hard:soft stoichiometry typically produces a diamondoid net, comprising alternating MN_4_ and MS_4_ tetrahedra (Figure 10). This structure type was first determined by Jeffery for CoHg(SCN)_4_,^20^ a compound historically widely used in microanalysis^212^ and as a calibrant for measures of magnetic susceptibility,^59^ though there is both a significant easy-plane zero field splitting, with *D* = +10.6(5) cm^–1^, and weak superexchange *zJ* = −0.2(1) cm^–1^.^61^ As this family crystallizes in the piezoelectric *I*4̅ space group, these compounds are also of interest for their nonlinear optical properties, particularly ZnHg(SCN)_4_,^39^ MnHg(SCN)_4_, and FeHg(SCN)_4_.^213^

The structure is now known for M[Hg(SCN)_4_] for M = Zn,^39^ Co,^20^ Fe,^213^ Mn,^213^ and Cd^214^ and Zn[Cd(SCN)_4_].^38,40^ Cu[Hg(SCN)_4_] adopts a related structure containing square planar Cu(NCS)_4_ ions. Cu[Hg(SCN)_4_] has been reported in which it has two subtly different polymorphs, a metrically orthorhombic monoclinic and true orthorhombic phase, which differ in the relative orientation of the square planar CuN_4_ coordination polyhedra and hence in topology: the orthorhombic phase has the 4,4T3 topology and the monoclinic phase has **pts** topology.^210,211,215,216^ Ni[Hg(SCN)_4_] is not known, with hydrates and methanolates forming instead (perhaps due to the difficulty of preparing tetrahedral Ni(II)).^217^ The crystal structure of a related composition, NiHg_2_(SCN)_6_, has been reported, but the unphysical NiN_6_ coordination sphere, with N–N distances as short as 1.64 Å and N–Ni–N angles of 50°, suggests the structure is incorrect.^218^ The true structure may be related to the complex structure of MnHg_2_(SCN)_6_ (section 3.4).^219^

### 3.3 3D: Other Structures

**Figure 11:**
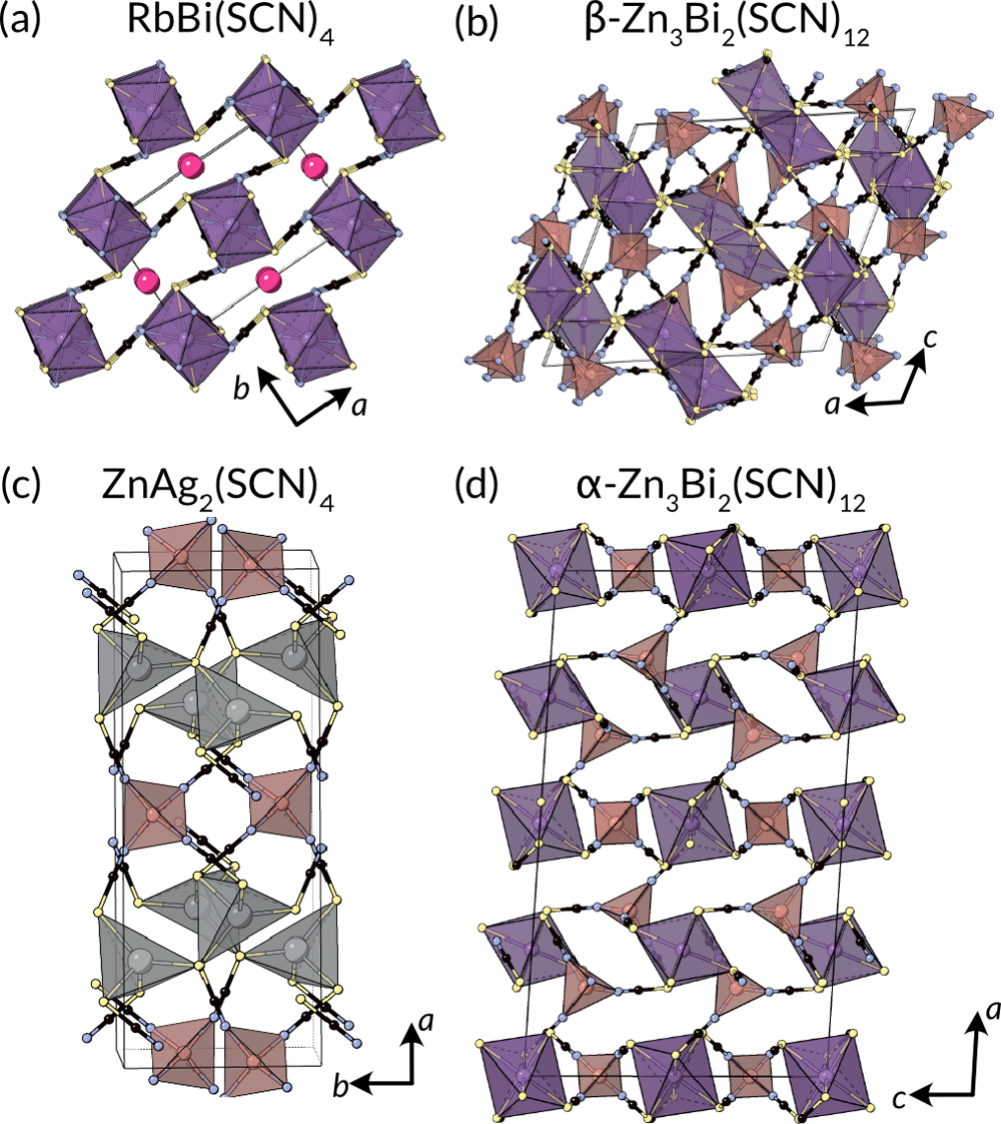
Structures of 3D ternaries. (a) Rb[Bi(SCN)_4_] has an anionic [Bi(SCN)_4_]^−^ framework,^220^ (b) β-Zn_3_Bi_2_NCS_6_ is assembled from BiS_6_ and ZnN_4_ polyhedra.^195^ (c) ZnAg(SCN)_4_ comprises ZnN_4_ and AgS_4_ tetrahedra, with the N terminus of NCS^–^ 1 coordinate and the S terminus 2 coordinate.^221,222^ (d) α-Zn_3_Bi_2_NCS_6_ consists of ZnBi(SCN)_4_ perovskite layers connected by Zn(NCS)_4_ tetrahedra.^195^

There a number of other more complex, 3D framework structures reported, formed from a mixture of tetrahedral and octahedral cations or other coordination geometries, though connections to the perovskitoid and diamondoid can be often be made (Figure 11). Zn_3_Bi_2_NCS_6_ is polymorphic, with both polymorphs assembled from ZnN_4_ tetrahedra and BiS_6_ octahedra connected by NCS^–^, with multiple symmetry-independent Bi and Zn sites in each structure. The topologies (**clw1** and **clw2**) of these two structures are otherwise unknown in the ToposPro database.^215^ α-Zn_3_Bi_2_NCS_6_ is of particular interest as it can be thought of as comprising Zn[Bi(SCN)_4_]-perovskite type layers, with every other layer containing an addition Zn_2_^+^ cation connecting them.^195^

Zn[Ag_2_(SCN)_4_] is assembled from corner-sharing ZnN_4_ and AgS_4_ tetrahedra which are connected through the length of the NCS ligands. However, whereas each N atom is only in one ZnN_4_ tetrahedron, each S atom is shared between two AgS_4_ tetrahedra, meaning NCS^–^ is three coordinate. The extended inorganic connectivity is reminiscent of semiconducting Cu(NCS).^221,222^

The RbBi(SCN)_4_ structure is related to both the perovskite and the postperovskite structures, with edge-sharing chains, corner sharing in the perpendicular directions to form a 3D network. The Rb^+^ cations sit within the pores of this structure.^220^ Literature reports make clear that many more 3D ternary thiocyanate frameworks are feasible.^66,223−229^

### 3.4 2D Layers

**Figure 12:**
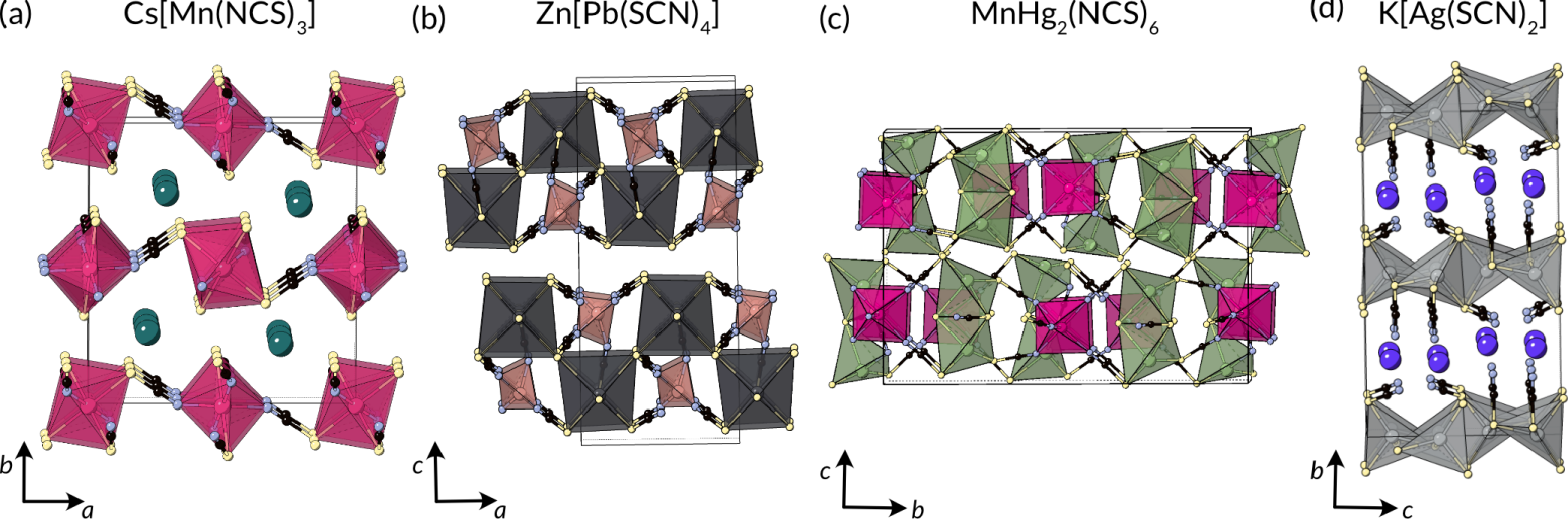
Structures of the layered ternary metal thiocyanates. (a) Postperovskite Cs[Mn(NCS)_3_],^63,230^ (b) Zn[Pb(SCN)_4_] with its {110} block perovskite structures,^21^ (c) MnHg_2_(NCS)_6_ with a complex, perovskite-related structure formed from MnN_6_ octahedra and Hg_2_ tetrahedral dimers,^219^ and (d) K[Ag(SCN)_2_] formed from edge- and corner-sharing AgS_4_ tetrahedra.^231,232^

There are a number of different ternary metal thiocyanates with primarily 2D connectivity (Figure 12). The most widely explored is the postperovskite structure, first reported for Rb[Cd(SCN)_3_]^83^ and later expanded to Cs[M(NCS)_3_], where M = Ni, Co, and Mn.^63,230^ These materials, like the perovskites, are formed from two different metal coordination environments, but in this case, they are MS_2_N_4_ and MS_4_N_2_. These polyhedra edge share through the length of the NCS^–^ ligand with polyhedra of the same kind and corner share with the others to form anionic sheets of composition [M(SCN)_3_]^−^ with the countercation lying between them. The postperovskite oxides and halides are typically formed between 5 and 100 GPa, e.g., MgSiO_3_,^233,234^ CaIrO_3_,^235^ and NaMF_3_ (M = Mg, Zn, Ni, Co, and Fe),^236−240^ and so the relative accessibility of thiocyanate postperovskites has allowed for more detailed investigation of their magnetism.^63^

Pb[Zn(NCS)_4_] comprises Pb(SCN)_6_ octahedra and Zn(NCS)_4_ tetrahedra, both significantly distorted and connected into neutral layers.^21^ Unlike the other discussed ternaries thus far, this compound contains extended Pb–S–Pb connections forming an infinite chain along the *b* axis. The PbS_2_ substructure resembles a 2D perovskite with {110}-type layers, e.g., the MnF_2_ substructure in BaMnF_4_.^241^

MnHg_2_(SCN)_6_ has a large number of distinct metal environments, with four different Hg sites and two different Mn sites.^219^ All Hg atoms form part of the Hg_2_(SCN)_6_ dimers of tetrahedra, with one dimer consisting of edge-sharing Hg(SCN)_4_ tetrahedra linked through the S atoms and the other containing one Hg(SCN)_4_ tetrahedron and one Hg(NCS)(SCN)_3_ tetrahedron edge sharing with one M–S–M bridge and one M–SCN–M bridge. One Mn is octahedrally coordinated, and the other has unusual square pyramidal coordination. The Hg_2_ dimers when considered as a unit are five or six coordinate, like the Mn ions, and are connected in an alternating fashion through the length of the thiocyanate ligand. The Hg_2_ and Mn coordination polyhedra edge share along the *c* axis but are corner sharing along the *a* axis. The five-coordinate square pyramidal Mn and the five-coordinated Hg_2_ dimer are aligned along the *a* axis and hence only have one-half the interchain connections due to their reduced coordination numbers. The layer structure can therefore be related to the M(NCS)_3_ layer postperovskite structure, though with considerable additional structural complexity.

K[Ag(SCN)_2_] is formed from AgS_4_ tetrahedra which edge share through the S atom to create dimers, and then, these dimers corner share through the S atom to form infinite anionic sheets. The N termini of the NCS^–^ anions point into the interlayer gallery toward the K atoms.^231,232^

### 3.5 1D Chains

**Figure 13:**
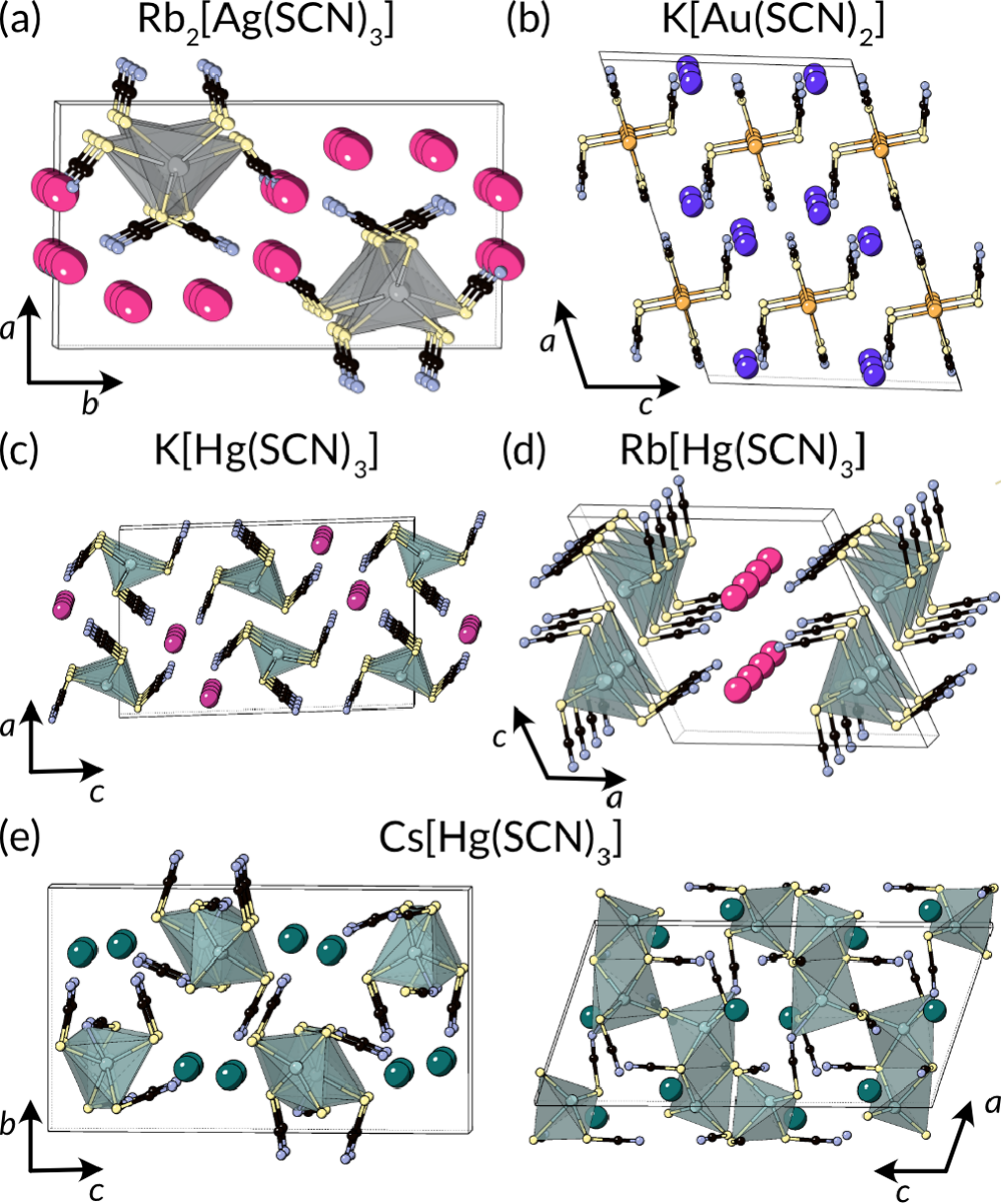
Structures of 1D ternary metal thiocyanates. (a) Rb_2_[Ag(SCN)_3_] comprising chains of corner-sharing AgS_4_ tetrahedra,^242^ (b) K[Au(SCN)_2_] consists of [Au(SCN)_2_]^−^ anions connected through strong aurophilic interactions,^243^ (c) K[Hg(SCN) and (d) Rb[Hg(SCN)_3_] also consist of chains of corner-sharing HgS_4_ tetrahedra.^83,84^ (e) Cs[Hg(SCN)_3_] contains edge-sharing dimerized tetrahedra connected through a Hg–NCS–Hg linkage.^83^

Low-coordinate soft metals can, in the right stoichiometry, form 1D structures with thiocyanate and alkali metals (Figure 13). Rb_2_[Ag(SCN)_3_] comprises a 1D chain of corner-sharing AgS_4_ tetrahedra.^242^ Similarly, both K[Hg(SCN)_3_] and Rb[Hg(SCN)_3_] consists of a chain of corner sharing HgS_4_ tetrahedra connected through the S atom of the NCS^–^ ligand, though the packing of these chains differs between the two compounds.^83,84^ Cs[Hg(SCN)_3_] also has a chain structure of tetrahedra, but in this case, the tetrahedra edge share through S to form Hg_2_S_6_N)_2_ dimers, which are connected through the length of thiocyanate to form extended 1D connectivity.^83^

The isostructural family M[Au(SCN)_2_], M = K, Rb, and Cs, has a very different kind of chain structure, comprising linearly coordinated Au(I) anions [Au(SCN)_2_]^−^ stacked and connected by aurophilic interactions, with a slight alternation of Au–Au distance, e.g., for K[Au(SCN)_2_] *d*_Au–Au_ = 3.04 and 3.00 Å.^243^ Different M cations lead to slightly different *d*_Au–Au_, which in turn produces differences in the optical fluorescence.

### 3.6 0D Salts

**Figure 14:**
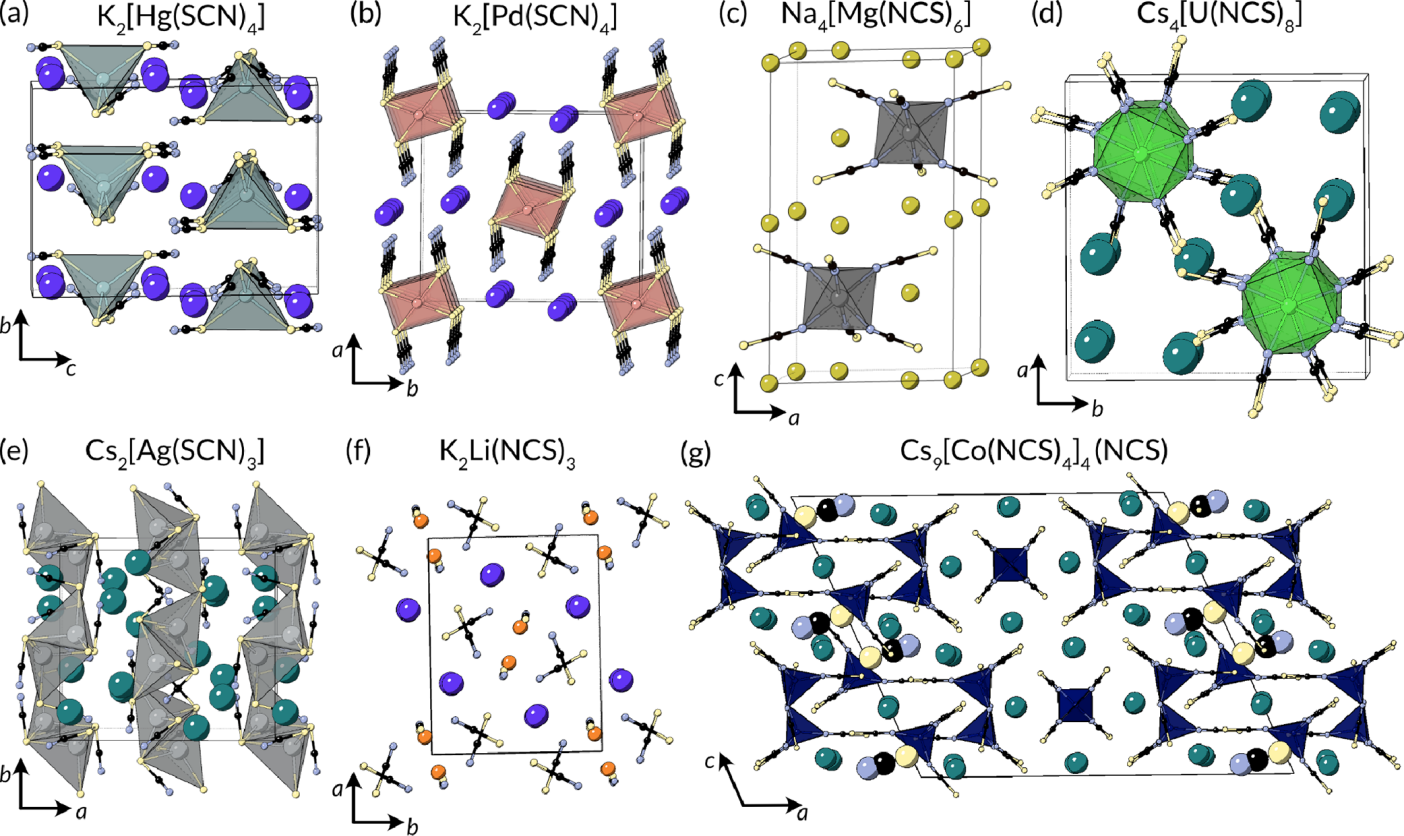
Structures of metal thiocyanate salts. Example of salts with each kind of local geometry are shown: (a) tetrahedral K_2_[Hg(SCN)_4_],^244^ (b) square planar K_2_[Pd(SCN)_4_],^245^ (c) octahedral Na_4_[Mg(SCN)_6_],^246^ (d) square antiprismatic Cs_4_[U(NCS)_8_],^247^ (e) tetrahedral dimers in Cs_2_[Ag(SCN)_3_],^242^ and mixed salts (f) K_2_Li(NCS)_3_^248^ and Cs_9_[Co(NCS)_4_]_4_(NCS), in which the nonbonded thiocyanate ion has been shown larger for clarity.^249^

The final class of ternaries are those with no extended coordination, which can be called salts, or vacancy-ordered derivatives of framework structures as appropriate (Figure 14). Coordination to alkali metals is neglected as the bonding is primarily ionic and nondirectional, and hence, their interactions with thiocyanate are typically not described as framework forming. However, this distinction is not always clear cut, and many thiocyanates not included in this section might also usefully be analyzed as ionic compounds. Anionic homoleptic metal thiocyanate complexes are probably the most diverse family of metal thiocyanate compounds, but reported crystal structures typically include organic countercations or solvent.^2,250,251^ There are thus surprisingly few crystal structures of purely inorganic ternary thiocyanate salts.

Most 0D metal thiocyanate structures have isolated metal centers, but there are two reported structures containing dimerized Ag(I) tetrahedra, Cs_2_[Ag(SCN)_3_] and K_2_[Ag(SCN)_3_], comprising edge-sharing AgS_4_ tetrahedra, stabilized by intradimer argentophilic interactions (*d*_Ag–Ag_ = 3.3 Å).^231,242^

The family of salts with the widest range of structures is that containing octahedrally coordinated metals, i.e., [M(NCS)*_x_*(SCN)_6–*x*_]^*y*−^ anions. Crystal structures of these octahedral coordination complexes have been reported for *y* = 2–4, and for *x* = 0 or 6, other values are reported with organic cations and solvates. Na_4_[Mg(NCS)_6_] has a trigonal structure, *P*3̅1*c*, and can be described by the alternation along the *c* axis of two different blocks, the first composed of [Mg(NCS)_6_]^4–^ and Na^+^ cations and the second containing just Na^+^ cations, though this is likely a very ionic structure.^246^ Cs_3_[Mo(NCS)_6_] is hexagonal, with columns of [Mo(NCS)_6_]^3–^ anions with Cs^+^ cations in between. The crystal structure shows very significant disorder which remains present down to 115 K.^252^ K_3_[Rh(SCN)_6_], despite the compositional similarity, adopts a different structure with isolated [Rh(SCN)_6_]^3–^ anions, but the structure has no information about the SCN^–^ ligand location due to limitations of contemporary crystallography.^253^ The limitations of historic crystallographic analysis unfortunately also affect K_2_[Pt(SCN)_6_] and Rb_2_[Pt(SCN)_6_], which are isostructural and crystallize in the space group *P*3̅*m*1.^199^ The thiocyanate anions cannot be resolved, but the arrangement of the alkali cations and [Pt(SCN)_6_]^2–^ anion, it is likely an *anti*-CdI_2_ structure type.^199^ The technical limitations and limited resolution of these older structures mean they must be treated with caution, and redetermination using modern instrumentation could likely uncover real chemical differences.

Next, there are four reported crystal structures with four-coordinate metals, tetrahedral K_2_[Hg(SCN)_4_]^244^ and K_3_[Ag(SCN)_4_]^231^ and the square-planar isostructural K_2_(Pd(SCN)_4_)^245^ and K_2_[Pt(SCN)_4_],^254^ both of which have weak axial M–S bonds which would connect the square planar units into chains.

The final family of coordination complexes is the eight-coordinate f-block metals. Cs_5_[Nd(NCS)_8_] has a NdN_8_ coordination geometry intermediate between cubic and square antiprismatic,^255^ whereas Cs_4_[U(NCS)_8_] has a square antiprismatic UN_8_ coordination.^247^ Both crystallize with isolated anions in tetragonal space groups: Nd in *I*4̅ and U in *P*4/*n*.

In addition to these simple salts, there are complex salts for which there are additional isolated thiocyanate ions alongside the thiocyanatometallate complexes. Cs_9_[Co(NCS)_4_]_4_(NCS) contains, in addition to the tetrahedral [Co(NCS)_4_]^2–^ anions which stack on top of either other, free NCS^–^ anions.^249^ K_2_Li(SCN)_3_ crystallizes in the polar *Pna*2_1_ space group, with the polarity largely arising from the arrangement of NCS^–^ anions. If coordination bonds between the alkali metals and NCS^–^ were considered, the structure could instead be thought of as a chain structure with LiN_3_S tetrahedra bridged into chains through the length of a NCS^–^ ligand. Each individual chain is polar, and each chain has the same polarity.^248^

## 4 Conclusion

The chemistry of known metal thiocyanates is very rich, and despite this richness, there are likely many more compounds to be discovered. The binary compounds presently known can be separated into five broad categories: the layered octahedral structures derived from the Ni(NCS)_2_ structure type; the distorted fluorite-type eight-coordinate structures analogous to Ba(NCS)_2_; the polymorphic tetrahedral structures found for Cu(I) and Ag(I); the ionic NaCl- and CsCl-derived structures found for group 1 and Tl^+^ thiocyanates; and the complex framework structures found for the d^10^ and p-block metals. These categories will likely become clearer as more structures are discovered, and there are 37 metal thiocyanates yet to be structurally characterized, of which 13 have not yet been reported at all.

Ternary thiocyanates naturally adopt a wider range of structures than the binaries. Two general themes are the prevalence of perovskitoid and diamondoid structures and the frequency of compositions combining soft and hard metals. There are undoubtedly many more ternary structures feasible, let alone quaternary or higher order compositions. The complexity of some of these structures is such that crystallography has been essential to determine the basic chemistries, with simple IR spectroscopic rules of thumb sometimes being misleading.

There are very few studies investigating the factors which determine why a given metal thiocyanate compound adopts the structure it does, but it is clear that ionicity, which favors dense structures (e.g., the alkali and alkali earth thiocyanates), softness, which determines whether thiocyanate binds through S or N, and the size of the ion, which is a key factor in the coordination number of the metal, are among the critical influences. Quantitative exploration of the relative stability of these structure types and the driving forces which lead to the observed structures will likely prove very valuable.

Making connections between molecular framework materials and inorganic frameworks built solely from individual atoms can be very productive, both to better understand existing molecular framework materials and to point the way toward potential new structure types that have not yet been realized. These analogies are of particular value because ab initio computational crystal structure prediction is not yet readily available for molecular frameworks: notably, none of the missing binary thiocyanates are present in recently reported extremely large data sets of hypothetical stable materials.^256−258^ This may well be because many molecular materials are thermodynamically metastable, despite their synthetic accessibility and kinetic stability. This review also highlights that the experimental literature continues to offer opportunities to discover new function through reinvestigation of known materials.^259^ For example, there are nine binary and ternary thiocyanates which are structurally two dimensional with neutral layers but which have not been investigated as potential monolayer materials (M(NCS)_2_, M = Cu, Ni, Co, Fe, Mn, Zn, Hg; ZnPb(NCS)_4_, and Mn_2_Hg(NCS)_6_), and more than one-half of these have not been previously proposed to be stable as monolayers.^256,260−262^ This suggests that the chemical insight and deep searching of the chemical literature can still be a fruitful route for uncovering brand new materials and useful functionality. In particular, although the majority of materials described in this review were described using salt metathesis, alternative synthetic routes, whether classical or using more modern techniques, could allow for the realization of presently unknown compounds.

This review builds on the book of Golub, Köhler, and Skopenko, last updated in 1986, and the structural understanding of this family has dramatically improved over the intervening decades due to the dedicated efforts of synthetic inorganic chemists and the significant advances in characterization. I anticipate that the continuing efforts of chemists, supported by the growing power of computation, means that a review of metal thiocyanates in 2060 will undoubtedly contain a much broader range of compounds and demonstrate deeper understanding of their structures and properties.

## Supporting Information Available

Table of compounds discussed in this review (PDF)
