# Supplementary material for: Inorganic Metal Thiocyanates
Source: Inorg Chem. 2024 Jul 9;63(29):13137–56. doi: 10.1021/acs.inorgchem.4c00920 (PMC11271006; doi:10.1021/acs.inorgchem.4c00920)
Supplement: Supplementary file 1 — ic4c00920_si_001.pdf [file ic4c00920_si_001.pdf]

Inorganic Metal Thiocyanates: Supporting Information

Matthew J. Cliffe *[a]

[a]School of Chemistry, University Park, Nottingham, NG7 2RD, United Kingdom

July 4, 2024

List of Tables

S1 Binary metal thiocyanates. 2
S2 Ternary metal thiocyanates reported in the ICSD as of May 2024. 4

*matthew.cliffe@nottingham.ac.uk

Table S1: Binary metal thiocyanates.

Compound	ICSD Code	Structure Type	Reference
Li(NCS)	425061	Na(NCS)	Reckeweg et al.[1]
$Be(NCS)_2$	-	Unknown	R. Buchner and Müller[2]
Na(NCS)	2005	Na(NCS)	Pistorius and Boeyens[3]
$Mg(NCS)_2$	-	Unknown	Joos et al.[4]
$Al(NCS)_3$	-	Unknown	Patel[5]
K(NCS)	36203	K(NCS)-LT	Klug[6]
K(NCS)	28356	K(NCS)-HT	Yamada and Watanabé[7]
$Ca(NCS)_2$	412783	$Sr(NCS)_2$	Wickleder and Larsen[8]
$Sc(NCS)_3$	-	Composition	Jiro Matsuura et al.[9]
$Ti(NCS)_4$	-	Composition	Böhland and Steinecke[10]
$V(NCS)_3$	-	Composition	Böhland and Zenker[11]
$Cr(NCS)_3$	-	Composition	Williams[12]
$Mn(NCS)_2$	101955	$Ni(NCS)_2$	Bassey et al.[13]; Neumann et al.[14]
$Fe(NCS)_2$		$Ni(NCS)_2$	Bassey et al.[13]
$Fe(NCS)_3$	-	Composition	Schlesinger and van. Valkenburgh[15]
$Co(NCS)_2$	250692	$Ni(NCS)_2$	Shurdha et al.[16]
$Ni(NCS)_2$	31320	$Ni(NCS)_2$	Dubler et al.[17]
$\alpha-$Cu(NCS)	124	α-Cu(NCS)	Kabesova et al.[18]
2 H$-\beta-$Cu(NCS)	32578	2H$-\beta$-Cu(NCS)	Smith and Saunders[19]
3 R$-\beta-$Cu(NCS)	24372	3R$-\beta$-Cu(NCS)	Smith and Saunders[20]
$Cu(NCS)_2$	131336	$Cu(NCS)_2$	Cliffe et al.[21]
$\alpha-Zn(NCS)_2$	-	Composition	Kynev and Dafinowa[22]
$\beta-Zn(NCS)_2$	22359	$Zn(NCS)_2$	Aslanov et al.[23]
$Ga(NCS)_3$	-	Composition	Patel and Tuck[24]
Rb(NCS)	116006	K(NCS)-LT	Shlyaykher et al.[25]
Rb(NCS)	116007	K(NCS)-HT	Shlyaykher et al.[25]
$Sr(NCS)_2$	94427	$Sr(NCS)_2$	Wickleder[26]
$Y(NCS)_3$	-	Composition	Slavkina et al.[27]
$Zr(NCS)_4$	-	Unknown	
$Nb(NCS)_5$	-	Composition	Böhland and Zenker[11]
$Mo(NCS)_x$	-	Unknown	
$Ru(NCS)_3$	-	Composition	Patel[28]
$Rh(NCS)_2$	-	Composition	Winkhaus and Ziegler[29]
$Rh(NCS)_3$	-	Composition	Patel[30]
$Pd(NCS)_2$	-	Composition	Sangari et al.[31]
$\alpha-$Ag(NCS)	16668	α-Ag(NCS)	Lindqvist[32]
$\beta-$Ag(NCS)	201359	β-Ag(NCS)	Smith et al.[33]
$Cd(NCS)_2$	31026	$Cd(NCS)_2$	Srdanov et al.[34]; Cannas et al.[35]
$In(NCS)_3$	-	Composition	Goggin et al.[36]

Compound	ICSD Code	Structure Type	Reference
$Sn(NCS)_2$	201193	$Sn(NCS)_2$	Chamberlain and Moser [37]
$Sn(NCS)_4$	-	Composition	Söderbäck [38]
$Sb(NCS)_3$	-	$Sb(NCS)_3$	Arlt et al. [39]
Cs(NCS)	19001	Cs(NCS)-LT	Manolatos et al. [40]
Cs(NCS)	-	Cs(NCS)-HT	Manolatos et al. [40]
$Ba(NCS)_2$	94428	$Sr(NCS)_2$	Wickleder [26]
$La(NCS)_3$	-	Composition	Slavkina et al. [27]
$Ce(NCS)_x$	-	Unknown	
$Pr(NCS)_3$	-	Composition	Slavkina et al. [27]
$Nd(NCS)_3$	-	Composition	Slavkina et al. [27]
$Sm(NCS)_3$	-	Composition	Slavkina et al. [27]
$Eu(NCS)_2$	94426	$Sr(NCS)_2$	Wickleder [26]
$Eu(NCS)_3$	-	Composition	Slavkina et al. [27]
$Gd(NCS)_3$	-	Composition	Slavkina et al. [27]
$Tb(NCS)_3$	-	Unknown	
$Dy(NCS)_3$	-	Composition	Slavkina et al. [27]
$Ho(NCS)_3$	-	Composition	Slavkina et al. [27]
$Er(NCS)_3$	-	Composition	Slavkina et al. [27]
$Tm(NCS)_3$	-	Unknown	
$Yb(NCS)_3$	-	Composition	Slavkina et al. [27]
$Lu(NCS)_3$	-	Composition	Slavkina et al. [27]
$Hf(NCS)_4$	-	Unknown	
$Ta(NCS)_5$	-	Composition	Böhland and Zenker [11]
$W(NCS)_x$	-	Unknown	
$Re(NCS)_4$	-	Composition	Hurd and Babler [41]; Geilmann et al. [42]
$Os(NCS)_x$	-	Unknown	
$Ir(NCS)_x$	-	Unknown	
$Pt(NCS)_2$	-	Composition	Yu. N. Kukushkin et al. [43]; Sangari et al. [31]
Au(NCS)	-	Composition	Gent and Gibson [44]
$Au(NCS)_3$	-	Composition	Williams [12]
Hg(NCS)	-	Composition	Dorm and Lindh [45]
$Hg(NCS)_2$	10304	$Hg(NCS)_2$	Beauchamp and Goutier [46]
Tl(NCS)	128322	K(NCS)-LT	Lippman and Rudman [47]
Tl(NCS)	128323	K(NCS)-HT	Lippman and Rudman [47]
$Pb(NCS)_2$	143	$Sr(NCS)_2$	Mokuolu and Speakman [48]
$Bi(NCS)_3$	9552	$Bi(NCS)_3$	Cliffe et al. [49]

Table S2: Ternary metal thiocyanates reported in the ICSD as of May 2024.

Compound	Dimensions	Subsection	ICSD code	Reference
$Cs_2Ag(SCN)_3$	0D	-	49620	Thiele and Kehr [50]
$K_2(Pt(SCN)_4)$	0D	-	69150	Hiltunen et al. [51]
$K_2Hg(SCN)_4$	0D	-	243965	Bandemehr et al. [52]
$K_2(Pd(SCN)_4)$	0D	-	9473	Mawby and Pringle [53]
$Na_4Mg(SCN)_6$	0D	-	94401	Wickleder and Larsen [54]
$K_3Rh(SCN)_6$	0D	-	20327	Zvonkova [55]
$Cs_3(Mo(NCS)_6)$	0D	-	69068	Delfs et al. [56]
$K_2(Pt(SCN)_6)$	0D	-	35511	Hendricks and Merwin [57]
$Rb_2(Pt(SCN)_6)$	0D	-	35512	Hendricks and Merwin [57]
$Cs_4(U(NCS)_8)$	0D	-	4104	Bombieri et al. [58]
$Cs_5(Nd(NCS)_8)$	0D	-	254486	Biswas et al. [59]
$Cs_9(Co(NCS)_4)_4(NCS)$	0D	-	33006	Byrne et al. [60]
$K_3(Ag(SCN)_4)$	0D	-	280726	Krautscheid and Gerber [61]
$K_2(Ag(SCN)_3)$	0D	-	280727	Krautscheid and Gerber [61]
$LiK_2(SCN)_3$	0D	-	430274	Reckeweg and DiSalvo [62]
$K(Hg(SCN)_3)$	1D	-	85761	Bowmaker et al. [63]
$Rb(Hg(SCN)_3)$	1D	-	101	Thiele and Messer [64]
$Cs(Hg(SCN)_3)$	1D	-	102	Thiele and Messer [64]
$Rb_2Ag(SCN)_3$	1D	-	49619	Thiele and Kehr [50]
$K(Au(SCN)_2)$	1D	-	159001	Coker et al. [65]
$Rb(Au(SCN)_2)$	1D	-	159002	Coker et al. [65]
$Cs(Au(SCN)_2)$	1D	-	159003	Coker et al. [65]
$Rb(Cd(SCN)_3)$	2D	-	14147	Thiele and Messer [64]
$CsNi(SCN)_3$	2D	-	59829	Fleck [66]
$K(Ag(SCN)_2)$	2D	-	280587	Krautscheid and Gerber [61]; Valkonen and Günes [67]
$PbZn(NCS)_4$	2D	-	62686	Brodersen et al. [68]
$Mn_2Hg_2(SCN)_6$	2D	-	163025	Li et al. [69]
$Co(Hg(SCN)_4)$	3D	diamondoid	36062	Jeffery [70]
$FeHg(SCN)_4$	3D	diamondoid	87888	Yunxing et al. [71]
$MnHg(SCN)_4$	3D	diamondoid	87889	Yunxing et al. [71]
$Cd(Hg(SCN)_4)$	3D	diamondoid	4102	Iizuka and Sudo [72]
$ZnHg(SCN)_4$	3D	diamondoid	280028	Xu et al. [73]
$ZnCd(SCN)_4$	3D	diamondoid	88970	Tian et al. [74]; Wang et al. [75]
orth-$Cu(Hg(SCN)_4)$	3D	diamondoid	28437	Porai-Koshits [76]
mono-$Cu(Hg(SCN)_4)$	3D	diamondoid	174608	Khandar et al. [77]
$RbBi(SCN)_4$	3D	other	9787	Gałdecki et al. [78]
$Zn(Ag(SCN)_2)_2$	3D	other	37160	Jones and Bembenek [79]; Lu et al. [80]
$\alpha{-}Zn_3(Bi(SCN)_6)_2$	3D	other	131337	Cliffe et al. [81]
$\alpha{-}Zn_3(Bi(SCN)_6)_2$	3D	other	131340	Cliffe et al. [81]
$CsCd(SCN)_3$	3D	perovskite	14148	Thiele and Messer [64]
$Mn(Pt(SCN)_6)$	3D	perovskite	5902	Tabe et al. [82]
$Mn(Pt(SCN)_6)$	3D	perovskite	5902	Tabe et al. [82]
$Sc(Bi(SCN)_6)$	3D	perovskite	9722	Cliffe et al. [49]
$Fe(Bi(SCN)_6)$	3D	perovskite	9740	Cliffe et al. [49]

References

[1] Reckeweg, O.; Schulz, A.; Blaschkowski, B.; Schleid, T.; DiSalvo, F. J. Single-Crystal Structures and Vibrational Spectra of Li[SCN] and Li[SCN]·$2H_2O$. *Zeitschrift für Naturforschung B* **2014**, *69b*, 17–24.

[2] R. Buchner, M.; Müller, M. Ethylenediamine Complexes of the Beryllium Halides and Pseudo-Halides. *Dalton Transactions* **2021**, *50*, 7246–7255.

[3] Pistorius, C. W. F. T.; Boeyens, J. C. A. Polymorphism and Melting of Sodium Cyanide and Thiocyanate to 40 kbar. *The Journal of Chemical Physics* **1968**, *48*, 1018–1022.

[4] Joos, M.; Conrad, M.; Merkle, R.; Schleid, T.; Maier, J.; Dinnebier, R. E.; Bette, S. Synthesis, Characterization and Thermal Behaviour of Solid Phases in the Quasi-Ternary System $Mg(SCN)_2$ - H_2O - THF. *Dalton Transactions* **2021**, *50*, 6949–6961.

[5] Patel, S. J. Aluminium(III) Isothiocyanate and Its Addition Compounds. *Journal of Inorganic and Nuclear Chemistry* **1971**, *33*, 17–22.

[6] Klug, H. P. The Crystal Structure of Potassium Thiocyanate. *Zeitschrift für Kristallographie - Crystalline Materials* **1933**, *85*, 214–222.

[7] Yamada, Y.; Watanabé, T. The Phase Transition of Crystalline Potassium Thiocyanate, KSCN. II. X-Ray Study. *Bulletin of the Chemical Society of Japan* **1963**, *36*, 1032–1037.

[8] Wickleder, C.; Larsen, P. $Ca(SCN)_2$ and $Ca(SCN)_2{\cdot}2H_2O$: Crystal Structure, Thermal Behavior and Vibrational Spectroscopy. *Zeitschrift für Naturforschung B* **2002**, *57*, 1419–1426.

[9] Jiro Matsuura,; Yasuhiro Karakawa,; Toru Murakami, Ether Extraction of Scandium Thiocyanate. *Bunseki Kagaku* **1965**, *14*, 267–269.

[10] Böhland, H.; Steinecke, H. Solvatfreies Titan(IV)-Thiocyanat Und Einige Abgeleitete Tetrathiocyanato-bis(Ligand)-Titan(IV)-Komplexe. *Zeitschrift fur anorganische und allgemeine Chemie* **1981**, *148*, 136–148.

[11] Böhland, H.; Zenker, E. Über Cyanatverbindungen Und Deren Reaktives Verhalten VI. Solvatfreies Niob(V)- Und Tantal(V)-Thiocyanat. *Journal of the Less Common Metals* **1968**, *14*, 397–402.

[12] Williams, H. E. *Cyanogen Compounds: Their Chemistry, Detection and Estmation*, 2nd ed.; Edward Arnold & Co: London, 1948.

[13] Bassey, E. N.; Paddison, J. A. M.; Keyzer, E. N.; Lee, J.; Manuel, P.; da Silva, I.; Dutton, S. E.; Grey, C. P.; Cliffe, M. J. Strengthening the Magnetic Interactions in Pseudobinary First-Row Transition Metal Thiocyanates, $M(NCS)_2$. *Inorganic Chemistry* **2020**, *59*, 11627–116339, Comment: 17 pages, 10 figures.

[14] Neumann, T.; Gallo, G.; Dinnebier, R. E.; Näther, C. Synthesis, Crystal Structures, and Properties of $Mn(NCS)_2$ Coordination Compounds with 4-Picoline as Coligand and Crystal Structure of $Mn(NCS)_2$. *Zeitschrift für anorganische und allgemeine Chemie* **2020**, *646*, 88–94.

[15] Schlesinger, H. I.; van. Valkenburgh, H. B. The Structure of Ferric Thiocyanate and the Thiocyanate Test for Iron. *Journal of the American Chemical Society* **1931**, *53*, 1212–1216.

[16] Shurdha, E.; Lapidus, S. H.; Stephens, P. W.; Moore, C. E.; Rheingold, A. L.; Miller, J. S. Extended Network Thiocyanate- and Tetracyanoethanide-Based First-Row Transition Metal Complexes. *Inorganic Chemistry* **2012**, *51*, 9655–9665.

[17] Dubler, E.; Relier, A.; Oswald, H. R. Intermediates in Thermal Decomposition of Nickel (II) Complexes : The Crystal Structures of $Ni(SCN)_2(NH_3)_2$ and Ni $(SCN)_2$. *Zeitschrift fur Kristallographie* **1982**, *161*, 265–277.

[18] Kabesova, M.; Dunaj-Jurco, M.; Serator, M.; Gazo, J.; Garaj, J. The Crystal Structure of Copper (I) Thiocyanate and Its Relation to the Crystal Structure of Copper (II) Diammine Dithiocyanate Complex. *Inorganica Chimica Acta* **1976**, *17*, 161–165.

[19] Smith, D. L.; Saunders, V. I. Preparation and Structure Refinement of the 2H Polytype of Beta-copper (I) Thiocyanate. *Acta Crystallographica* **1982**, *B38*, 907–9.

[20] Smith, D. L.; Saunders, V. I. The Structure and Polytypism of the β Modification of Copper(I) Thiocyanate. *Acta Crystallographica* **1981**, *B37*, 1807–1812.

[21] Cliffe, M. J.; Lee, J.; Paddison, J. A. M.; Schott, S.; Mukherjee, P.; Gaultois, M. W.; Manuel, P.; Sirringhaus, H.; Dutton, S. E.; Grey, C. P. Low-Dimensional Quantum Magnetism in $Cu(NCS)_2$: A Molecular Framework Material. *Physical Review B* **2018**, *97*, 144421.

[22] Kynev, K.; Dafinowa, R. Polymorphie Und Lumineszenz Der Zinkrhodanid-Luminophore. *Comptes rendus de l'Academi bulgare des Sciences* **1967**, *20*, 939–942.

[23] Aslanov, L. A.; Ionov, V. M.; Kynev, K. Crystal-Structure of Anhydrous Zinc Rhodanide. *Crystallography reports* **1976**, *21*, 1198–1199.

[24] Patel, S. J.; Tuck, D. G. Gallium(III) Isothiocyanate and Its Addition Compounds. *Canadian Journal of Chemistry* **1969**, *47*, 229–233.

[25] Shlyaykher, A.; Pippinger, T.; Schleid, T.; Reckeweg, O.; Tambornino, F. Syntheses, Crystallographic Characterization, and Structural Relations of Rb[SCN]. *Zeitschrift für Kristallographie - Crystalline Materials* **2022**, *237*, 69–75.

[26] Wickleder, C. $M(SCN)_2$ (M = Eu , Sr , Ba): Kristallstruktur , Thermisches Verhalten , Schwingungsspektroskopie $M(SCN)_2$ (M = Eu, Sr ,Ba): Crystal Structure, Thermal Behaviour, Vibrational. *Zeitschrift Fur Anorganische Und Allgemeine Chemie* **2001**, *627*, 1693.

[27] Slavkina, R. I.; Usubaliev, D.; Serebrennikov, V. V. Preparation of Anhydrous Perchlorates and Thiocyanates of Some Rare Earth Elements. *Tr. Tomskogo Gos. Univ., Ser. Khim.* **1963**, *157*, 304–306.

[28] Patel, S. J. The Synthesis of Anhydrous Ruthenium(III) Isothiocyanate and Its Monohydrate. *Boletin de la Sociedad Chilena de Quimica* **1972**, *19*, 13–14.

[29] Winkhaus, G.; Ziegler, P. Rhodium(II)-Halogenacetatokomplexe. *Zeitschrift für anorganische und allgemeine Chemie* **1967**, *350*, 51–61.

[30] Patel, S. J. The Synthesis of Anhydrous Rhodium(III) Thiocyanate Using Non-Aqueous Media. *Boletin de la Sociedad Chilena de Quimica* **1972**, *19*, 15–16.

[31] Sangari, H. S.; Sodhi, G. S.; Kaur, J. Thermal Studies on Platinum Metal Complexes of N-methylcyclohexyl Dithiocarbamate. *Thermochimica Acta* **1990**, *171*, 49–55.

[32] Lindqvist, I. On the Crystal Structure of Silver Thiocyanate. *Acta Crystallographica* **1957**, *10*, 29–32.

[33] Smith, D. L.; Maskasky, J. E.; Spaulding, L. R. Polymorphism in Silver Thiocyanate: Preparation of a New Phase and Its Characterization by X-ray Powder Diffraction. *Journal of Applied Crystallography* **1982**, *15*, 488–492.

[34] Srdanov, G.; Herak, R.; Prelesnik, B.; Jeremic, M. Crystal Structure of Cadmium(II) Thiocyanate. *Bulletin de la Société Chimique Beograd* **1979**, *44*, 561–565.

[35] Cannas, M.; Carta, G.; Cristini, A.; Marongiu, G. Three-Co-Ordinate Thiocyanate in Cadmium Dithiocyanate. *Journal of the Chemical Society, Dalton Transactions* **1976**, 300–301.

[36] Goggin, P. L.; McColm, I. J.; Shore, R. Indium Tricyanide and Indium Trithiocyanate. *Journal of the Chemical Society A: Inorganic, Physical, Theoretical* **1966**, 1314.

[37] Chamberlain, B. R.; Moser, W. Tin(II) Thiocyanate and Complex Thiocyanates. *Journal of the Chemical Society A: Inorganic, Physical, Theoretical* **1969**, *0*, 354–358.

[38] Söderbäck, E. Studien Über Das Freie Rhodan. *Justus Liebigs Annalen der Chemie* **1919**, *419*, 217–322.

[39] Arlt, S.; Harloff, J.; Schulz, A.; Stoffers, A.; Villinger, A. Heavy Neutral and Anionic Pnictogen Thiocyanates. *Inorganic Chemistry* **2019**, *58*, 5305–5313.

[40] Manolatos, S.; Tillinger, M.; Post, B. Polymorphism in Cesium Thiocyanate. *Journal of Solid State Chemistry* **1973**, *7*, 31–35.

[41] Hurd, L. C.; Babler, B. J. The Determination of Rhenium. *Industrial & Engineering Chemistry Analytical Edition* **1936**, *8*, 112–114.

[42] Geilmann, W.; Wrigge, F. W.; Weibke, F. Beiträge zur analytischen Chemie des Rheniums. 7. Der Nachweis und die Bestimmung Kleiner Rheniummengen mit Hilfe von Kali-

umrhodanid und Zinnchlorür. *Zeitschrift für anorganische und allgemeine Chemie* **1932**, *208*, 217–224.

[43] Yu. N. Kukushkin,; L. V. Vrublevskaya,; S. I. Bakhireva,; E. N. Kalyukova, Thermal Transformation of Platinum Thiocyanato- and Cyano-complexes of the Magnus' Salt Type. *Russian Journal of Inorganic Chemistry* **1980**, *25*, 723–726.

[44] Gent, W. L. G.; Gibson, C. S. 393. The Organic Compounds of Gold. Part XI. Diethylthiocyanatogold. *Journal of the Chemical Society (Resumed)* **1949**, 1835–1840.

[45] Dorm, E.; Lindh, B. Preparative and X-Ray Diffraction Studies on Several Mercury(I) Salts. *Acta Chemica Scandinavica* **1967**, *21*, 1661–1662.

[46] Beauchamp, A. L.; Goutier, D. Structure Cristalline et Moléculaire Du Thiocyanate Mercurique. *Canadian Journal of Chemistry* **1972**, *50*, 977–981.

[47] Lippman, R.; Rudman, R. Dynamic Twinning: Polymorphism of Thallium Thiocyanate Studied with X-ray Diffraction. *The Journal of Chemical Physics* **1983**, *79*, 3457–3461.

[48] Mokuolu, J. A. A.; Speakman, J. C. The Crystal Structure of Lead(II) Thiocyanate. *Acta Crystallographica* **1975**, *B31*, 172–176.

[49] Cliffe, M. J.; Keyzer, E. N.; Dunstan, M. T.; Ahmad, S.; De Volder, M. F. L.; Deschler, F.; Morris, A. J.; Grey, C. P. Strongly Coloured Thiocyanate Frameworks with Perovskite-Analogue Structures. *Chemical Science* **2019**, *10*, 793–801.

[50] Thiele, G.; Kehr, W. Über die Trithiocyanatoargentate $Rb_2Ag(SCN)_3$ und $Cs_2Ag(SCN)_3$. *Zeitschrift für anorganische und allgemeine Chemie* **1984**, *515*, 199–206.

[51] Hiltunen, L.; Hölsä, J.; Strek, W. Crystal Structure and Thermal Stability of Potassium Tetrathiocyanatoplatinate(II), $K_2Pt(SCN)_4$. *Inorganica Chimica Acta* **1990**, *178*, 243–248.

[52] Bandemehr, J.; Conrad, M.; Kraus, F. Redetermination of the Crystal Structure of $K_2Hg(SCN)_4$. *Acta Crystallographica* **2017**, *E73*, 1073–1075.

[53] Mawby, A.; Pringle, G. E. The Structure of Potassium Tetrathiocyanatopalladate(II). *Journal of Inorganic and Nuclear Chemistry* **1972**, *34*, 2213–2217.

[54] Wickleder, C.; Larsen, P. BaClSCN und $Na_4Mg(SCN)_6$: Zwei neue wasserfreie Thiocyanate der Erdalkalimetalle. *Zeitschrift für anorganische und allgemeine Chemie* **2001**, *627*, 1279–1282.

[55] Zvonkova, Z. V. The Crystal Structure of Rhodates. 8. The Crystal Structure of the Complex Rhodium Hexarhodanites. *Zhurnal Fizicheskoi Khimii* **1953**, *27*, 100–105.

[56] Delfs, C. D.; Figgis, B. N.; Kucharski, E. S.; Reynolds, P. A. Charge Density in the $[Mo(NCS)_6]^{3-}$ Ion in $Cs_3Mo(NCS)_6$. *Journal of the Chemical Society, Dalton Transactions* **1989**, 1779–1785.

[57] Hendricks, S. B.; Merwin, H. E. The Atomic Arrangement in Crystals of the Alkali Platini-Thiocyanates. *American Journal of Science* **1928**, *15*, 487–494.

[58] Bombieri, G.; Moseley, P. T.; Brown, D. Crystal Structure of Tetracaesium Octaisothiocyanatouranate(IV). *Journal of the Chemical Society, Dalton Transactions* **1975**, 1520–1523.

[59] Biswas, S.; Ma, S.; Nuzzo, S.; Twamley, B.; Russell, A. T.; Platts, J. A.; Hartl, F.; Baker, R. J. Structural Variability of 4f and 5f Thiocyanate Complexes and Dissociation of Uranium(III)–Thiocyanate Bonds with Increased Ionicity. *Inorganic Chemistry* **2017**, *56*, 14426–14437.

[60] Byrne, N. M.; Schofield, M. H.; Nicholas, A. D.; Cahill, C. L. Bimetallic Uranyl/Cobalt(Ii) Isothiocyanates: Structure, Property and Spectroscopic Analysis of Homo- and Heterometallic Phases. *Dalton Transactions* **2021**, *50*, 9158–9172.

[61] Krautscheid, H.; Gerber, S. Potassium Thiocyanate Argentates: $K_3[Ag(SCN)_4]$, $K_4[Ag_2(SCN)_6]$ and $K[Ag(SCN)_2]$. *Acta Crystallographica* **2001**, *C57*, 781–783.

[62] Reckeweg, O.; DiSalvo, F. J. The First Pseudo-Ternary Thiocyanate Containing Two Alkali Metals – Synthesis and Single-Crystal Structure of $LiK_2[SCN]_3$. *Zeitschrift für Naturforschung B* **2016**, *71*, 161–164.

[63] Bowmaker, G. A.; Churakov, A. V.; Harris, R. K.; Howard, J. A. K.; Apperley, D. C. Solid-State ^{199}Hg MAS NMR Studies of Mercury (II) Thiocyanate Complexes and Related Compounds. Crystal Structure of $Hg(SeCN)_2$. *Inorganic Chemistry* **1998**, *37*, 1734–1743.

[64] Thiele, G.; Messer, D. Die Kristallstrukturen der Trithiocyanatomercurate $RbHg(SCN)_3$ und $CsHg(SCN)_3$. *Zeitschrift für anorganische und allgemeine Chemie* **1976**, *421*, 24–36.

[65] Coker, N. L.; Krause Bauer, J. A.; Elder, R. C. Emission Energy Correlates with Inverse of Gold-Gold Distance for Various $[Au(SCN)_2]^-$ Salts. *Journal of the American Chemical Society* **2004**, *126*, 12–13.

[66] Fleck, M. Thiocyanates of Nickel and Caesium: $Cs_2NiAg_2(SCN)_6.2H_2O$ and $CsNi(SCN)_3$. *Acta Crystallographica* **2004**, *C60*, i63–i65.

[67] Valkonen, J.; Günes, M. Potassium Silver Thiocyanate. *Acta Crystallographica* **2001**, *E57*, i52–i54.

[68] Brodersen, K.; Procher, H.; Hummel, H.-U. Zur Darstellung Und Kristallstruktur $PbZn(NCS)_4$. *Zeitschrift für Naturforschung B* **1987**, *42*, 679–681.

[69] Li, G.-H.; Fang, Q.; Xue, G.; Yu, W.-T. Synthesis and Crystal Structure of New Coordination Compound $Mn_2Hg_4(SCN)_{12}$. *Chinese Journal of Inorganic Chemistry* **2005**, *21*, 91–94.

[70] Jeffery, J. W. Crystal Structure of $Co[Hg(CNS)_4]$. *Nature* **1947**, *159*, 610–610.

[71] Yunxing, Y.; Fang, Q.; Yuan, D.; Tian, Y.; Jiang, M.; Williams, I. D.; Zhigang, C. Synthesis, Structure and Non-Linear Optical Properties of $FeHg(SCN)_4$ and $MnHg(SCN)_4$. *Acta Chimica Sinica* **1999**, *57*, 1257–1261.

[72] Iizuka, M.; Sudo, T. Crystal Structure of Cadmium Mercuric Thiocyanate, $CdHg(SCN)_4$. *Zeitschrift für Kristallographie* **1968**, *126*, 376–378.

[73] Xu, D.; Yu, W.-T.; Wang, X.-Q.; Yuan, D.-R.; Lu, M.-K.; Yang, P.; Guo, S.-Y.; Meng, F.-Q.; Jiang, M.-H. Zinc Mercury Thiocyanate (ZMTC). *Acta Crystallographica* **1999**, *C55*, 1203–1205.

[74] Tian, Y.-P.; Yu, W.-T.; Fang, Q.; Wang, X.-Q.; Yuan, D.-R.; Xu, D.; Jiang, M.-H. Zinc Cadmium Thiocyanate (ZCTC). *Acta Crystallographica* **1999**, *C55*, 1393–1395.

[75] Wang, X. Q.; Xu, D.; Yuan, D. R.; Tian, Y. P.; Yu, W. T.; Sun, S. Y.; Yang, Z. H.; Fang, Q.; Lu, M. K.; Yan, Y. X.; Meng, F. Q.; Guo, S. Y.; Zhang, G. H.; Jiang, M. H. Synthesis, Structure and Properties of a New Nonlinear Optical Material: Zinc Cadmium Tetrathiocyanate. *Materials Research Bulletin* **1999**, *34*, 2003–2011.

[76] Porai-Koshits, M. A. The Structural Motifs of the Crystals of Some Thiocyanato Compounds of Divalent Nickel and Copper. *Journal of Structural Chemistry* **1963**, *4*, 531–539.

[77] Khandar, A. A.; Klein, A.; Bakhtiari, A.; Mahjoub, A. R.; Pohl, R. W. H. Dicarboxylate Assisted Synthesis of the Monoclinic Heterometallic Tetrathiocyanato Bridged Copper(II) and Mercury(II) Coordination Polymer $\{Cu[Hg(SCN)_4]\}_n$. *Journal of Solid State Chemistry* **2011**, *184*, 379–386.

[78] Gałdecki, Z.; Główka, M. L.; Goliński, B. The Crystal and Molecular Structure of Rubidium Tetrathiocyanatobismuthate(III). *Acta Crystallographica* **1976**, *B32*, 2319–2321.

[79] Jones, P. G.; Bembenek, E. Redetermination of the Structure of Zinc Disilver(I) Tetrathiocyanate (at 178 K). *Acta Crystallographica* **1992**, *C48*, 1361–1362.

[80] Lu, S.-F.; He, M.-Y.; Huang, J.-L. The Crystal Structure of Bimetallic Zinc(II) Silver(I) Tetrathiocyanate Complex $ZnAg_2(SCN)_4$. *Chinese Journal of Structural Chemistry* **1982**, *1*, 72–76.

[81] Cliffe, M. J.; Keyzer, E. N.; Bond, A. D.; Astle, M. A.; Grey, C. P. The Structures of Ordered Defects in Thiocyanate Analogues of Prussian Blue. *Chemical Science* **2020**, *11*, 4430–4438.

[82] Tabe, H.; Matsushima, M.; Tanaka, R.; Yamada, Y. Creation and Stabilisation of Tuneable Open Metal Sites in Thiocyanato-Bridged Heterometallic Coordination Polymers to Be Used as Heterogeneous Catalysts. *Dalton Transactions* **2019**, *48*, 17063–17069.
